# Molecular Design Strategies for Electrochemical Behavior of Aromatic Carbonyl Compounds in Organic and Aqueous Electrolytes

**DOI:** 10.1002/advs.201900431

**Published:** 2019-07-25

**Authors:** Huiling Peng, Qianchuan Yu, Shengping Wang, Jeonghun Kim, Alan E. Rowan, Ashok Kumar Nanjundan, Yusuke Yamauchi, Jingxian Yu

**Affiliations:** ^1^ Faculty of Materials Science and Chemistry China University of Geosciences Wuhan 430074 China; ^2^ Key Laboratory of Eco‐chemical Engineering College of Chemistry and Molecular Engineering Qingdao University of Science and Technology Qingdao 266042 China; ^3^ Australian Institute for Bioengineering and Nanotechnology (AIBN) The University of Queensland Brisbane QLD 4072 Australia; ^4^ School of Chemical Engineering Faculty of Engineering Architecture and Information Technology (EAIT) The University of Queensland Brisbane QLD 4072 Australia; ^5^ International Center for Materials Nanoarchitectonics (MANA) National Institute for Materials Science (NIMS) 1‐1 Namiki Tsukuba Ibaraki 305‐0044 Japan; ^6^ ARC Centre of Excellence for Nanoscale BioPhotonics (CNBP) School of Chemistry and Physics The University of Adelaide Adelaide SA 5005 Australia

**Keywords:** aromatic carbonyl compounds, batteries, electrochemical behavior, electrode materials

## Abstract

To sustainably satisfy the growing demand for energy, organic carbonyl compounds (OCCs) are being widely studied as electrode active materials for batteries owing to their high capacity, flexible structure, low cost, environmental friendliness, renewability, and universal applicability. However, their high solubility in electrolytes, limited active sites, and low conductivity are obstacles in increasing their usage. Here, the nucleophilic addition reaction of aromatic carbonyl compounds (ACCs) is first used to explain the electrochemical behavior of carbonyl compounds during charge–discharge, and the relationship of the molecular structure and electrochemical properties of ACCs are discussed. Strategies for molecular structure modifications to improve the performance of ACCs, i.e., the capacity density, cycle life, rate performance, and voltage of the discharge platform, are also elaborated. ACCs, as electrode active materials in aqueous solutions, will become a future research hotspot. ACCs will inevitably become sustainable green materials for batteries with high capacity density and high power density.

## 1 Introduction

Energy depletion and environmental pollution have seriously restricted economic development. The current energy storage and power problems that need to be solved include a higher energy density, a higher power density, a longer cycle life, and a lower self‐discharge rate of chemical power supplies.^1^, ^2^, ^3^, ^4^, ^5^, ^6^, ^7^, ^8^, ^9^ However, the commercialization of inorganic electrode materials is limited by the capacity density and energy density, and battery systems containing Fe_3_O_4_,^10^ LiFePO_4_,^11^ LiCoO_2_,^12^, ^13^ LiMn_2_O_4_,^14^ Li_4_Ti_5_O_12_,^15^ and organic electrochemically active molecules as electrode materials have been rapidly developed.^16^, ^17^, ^18^, ^19^ As electrode active materials, organic molecules have the advantages of structure adjustment, reactive active sites, having renewable resources, and redox stability, and their multielectron, multistep oxidation/reduction can provide high capacity density.^20^, ^21^, ^22^ On the one hand, aromatic carbonyl compounds (ACCs) can be used in both aqueous and organic solution electrolytes in batteries. On the other hand, organic electrode materials are being widely explored for various metal ion batteries, including lithium‐ion batteries (LIBs), sodium‐ion batteries (SIBs), potassium‐ion batteries (KIBs), magnesium‐ion batteries (MIBs), zinc‐ion batteries (ZIBs), aluminum‐ion batteries (AIBs), and calcium‐ion batteries (CIBs).^23^, ^24^, ^25^

Recently studied electrochemically active organic materials can be classified into different categories, such as conductive polymer organic sulfur compounds, azo compounds, heterocyclic‐based compounds, organic radical compounds, and ACCs.^26^, ^27^, ^28^, ^29^ Among them, ACCs exhibiting a stable electrochemical reaction mechanism, are widely regarded as the most promising next‐generation electrode materials. ACCs are classified into four types according to the position of the carbonyl group in the active center: carboxylate, imide, quinone, or ketone.^30^, ^31^, ^32^, ^33^, ^34^ In contrast to inorganic materials and other organic compounds, ACCs are mainly derived from natural biomass, thus sources are renewable and environmentally friendly.^35^, ^36^, ^37^, ^38^, ^39^ Their components are mainly C, H, O, N, S, and other elements with lower density, which is beneficial for increasing the capacity density.^40^ Their flexible molecular structure can accommodate more Li^+^ and allow for ultrafast (dis) charge kinetics.^41^, ^42^ Such a molecular structure with special electrochemical properties can be obtained via a mild synthesis route through the controllable adjustment of the molecular structure.^43^, ^44^, ^45^, ^46^ The reaction mechanism of ACCs for various cations in organic and aqueous electrolytes exhibits universal applicability.^40^, ^46^, ^47^, ^48^ However, high solubility, low conductivity, and low active carbonyl utilization are the major obstacles for further research and development of ACCs.^49^, ^50^ Fortunately, the flexible structure and controllable adjustment of ACCs can help modify the molecular structure, which underlines the influence of the molecular structure on electrochemical performance; these properties can also be used to guide the design of carbonyl molecules with special electrochemical properties.^3^ Recently, Chen et al.^51^ has demonstrated that ACCs can be modified, and they suggest molecular engineering approaches via changes in electrochemical properties. Schubert et al.^52^ reported the influence of different molecular structure, conductive agents, and binders on battery performance. They classified carbonyl compounds according to the function of the electrode (positive/negative/double electrode), and the unique conjugated structure and design flexibility of the compound containing the polymerized conjugated structure were elaborated.^53^ Chen et al. reported that the dissolution of low molecular weight ACCs could be alleviated by limiting the voltage range, fixation onto insoluble substrates, and choosing a suitable electrolyte and that the conductivity of nanocomposites could be improved by compounding with carbon nanotube (CNT) and graphene oxide (GO).^54^ Chou et al.^55^ reported that organic electrode materials can restrain solubility, improve the voltage of the discharge platform (*V*_platform_), and increase conductivity using polymerization reactions, salification, electrolyte selection, binders, and conductive agents. Later, Chen et al.^40^ used structural stability as a criterion to distinguish the redox reaction mechanisms and classified them according to electrochemical parameters such as capacity, potential, kinetics, stability, etc. They also highlighted a molecular modification method for structural adjustment under different electrochemical parameters to predict high energy density, high power density, safety, and long cycle‐stable organic electrode materials. The starting point of this review is to explain the advantages and disadvantages of the ACC materials currently used in organic electrolytes and aqueous electrolytes according to their molecular structure and to describe molecular design principles for optimizing electrical properties and for obtaining the most advantageous molecular structure size. It is predicted that ACC materials will replace inorganic materials in the field of energy storage materials.

ACCs exhibit bond fracture and reconstruction during charge–discharge and undergo redox reactions with cations. This can be understood as the combination and fracture of O, containing a lone pair of electrons, in the carbonyl group and a metal ion having an empty orbital. The reaction appears as a nucleophilic addition, consisting of a deformation reaction of the carbonyl and enolized structures. During the discharge process, electrons move from the negative and positive electrodes, and the carbonyl group accepts an e^−^ to form a first‐order negative ion. To maintain electrical neutrality of the system, the first‐order negative ion forms an O—Li bond with the free Li^+^ in the electrolyte.^56^ The carbonyl activity can be predicted and judged by nucleophilic addition activity. However, some materials not only have electrochemical battery behavior based on nucleophilic addition reactions, they also show the phenomenon of pseudocapacitance behavior, which can contribute some capacity.^57^, ^58^ The electrochemical reaction mechanism of ACCs in secondary batteries has only recently been explained by nucleophilic addition. The electrochemical properties have been predicted according to the activity and stability of the active sites in the molecular structure. ACC material is suitable for many types of cationic batteries. In organic electrolytes, the application of ACCs in LIBs is mainly studied because of the harsh manufacturing conditions and complex processes of electrolytes. A series of laws of the influence of structure on performance have been obtained, and many potential molecular structures are speculated. In contrast, the low impact of aqueous electrolytes on the environment enable many types of cationic batteries to be explored simultaneously, which has resulted in many new possibilities in this area. This research has also revealed differences in the reaction mechanism and storage capacity of cationic ions with different radii. The exploration of different cations in aqueous electrolytes can guide researchers to use different cations selectively to relieve the pressure of metal energy.

## 2 Electrochemical Reaction Mechanisms of ACCs

### 2.1 Nucleophilic Addition Mechanism

#### 2.1.1 Nucleophilic Addition Reaction of ACCs

The length of the active center for the nucleophilic addition reaction is ≈0.122 nm. Since the electronegativity (3.5) of O is greater than that of C (2.5), C in the carbonyl group is positively charged and O is negatively charged. In organic chemistry, the nucleophilic addition reaction of carbonyl groups is catalyzed by acids and alkalis, depending on the solution environment. In acid‐catalyzed reactions, the nucleophilic reagents attack oxygen in the carbonyl group first, whereas the alkali‐catalyzed nucleophilic reaction attacks the carbon in the carbonyl group. The rate‐limiting step is the carbon attack.

**Figure 1:**
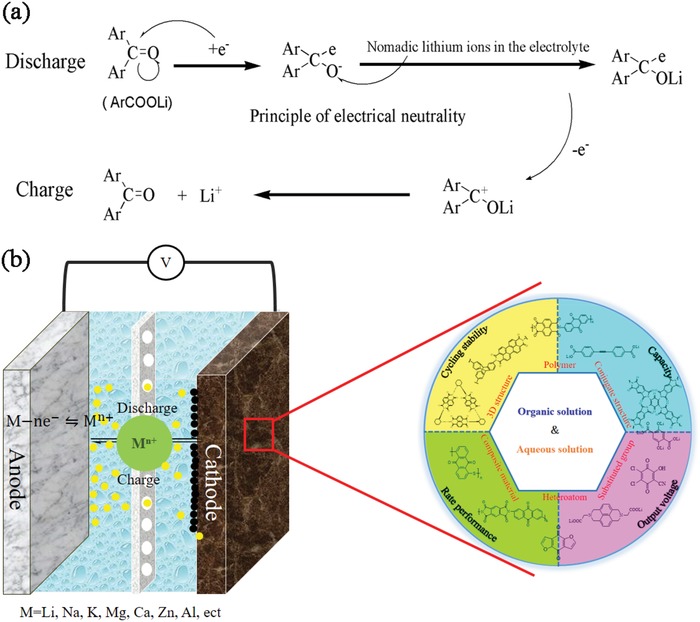
a) Reaction formula of ACCs in LIBs. b) The cell configurations and charge transfer processes in MIBs.

The solvents in organic electrolytes are mainly ethers and esters, and the nucleophilic addition reaction of carbonyl groups occurs under base alkaline environments. The conversion process of the enolization structure involves a charge transfer, which exhibits good electrochemical behavior. This reversible electrochemical reaction can generally be divided into two stages (**Figure** **1**a): 1) ACCs form oxygen anions. Electrons then attack the carbonyl carbon to form a first‐order anion, R—C=O → R—C—O; 2) unstable oxygen in the first‐order anion combines with Li^+^ in the electrolyte to neutralize the charge, R—C—O^. .^→ R—C—O—Li.

The structure of the electrochemical active center is summarized as follows. 1) The number of active carbonyl groups is proportional to the theoretical capacity density (*C*_theoretical_) and the difficulty of the reaction. 2) The ordered conjugated structure can improve the planarization of the molecular structure, reduce the activation energy of the nucleophilic addition reaction, and increase the *V*_platform_. 3) The electron withdrawing groups can improve the electron cloud density of the carbonyl carbon, speed up the reaction, and improve the multiplying performance. 4) Heteroatoms that are more electronegative than carbon, such as O, N, and S, can promote nucleophilic addition.

#### 2.1.2 Structural Characteristics of ACCs

A more stable structure of ACCs is more favorable to stabilize the nucleophilic addition reaction of the sp^2^ to sp^3^ transition of the carbonyl groups in the battery system. Organic carbonyl compounds (OCCs), can be classified into chain carbonyl compounds (CCCs) and ACCs. Most CCCs are liquids with a certain volatility and high solubility in electrolytes, exhibiting a single electron nucleophilic addition reaction. CCCs are more suitable as electrode materials for liquid flow batteries. The formation of the delocalized π bond of the ACCs results in the formation of a conjugate plane to average the bond length, reduces the lowest unoccupied molecular orbital (LUMO) energy, and enhances the stability of the molecule. The nucleophilic addition reaction of the two electrons can contribute to structural stability and charge balance. ACCs are solid at room temperature and are insoluble in organic electrolytes. Therefore, the structural stability of ACCs is better than that of other non‐ACC carbonyl compounds and other organic compounds. First, having a larger conjugated molecular structure can make the molecules have better planarity and more stable carbonyl molecules. Second, lower molecular energy can provide highly active carbonyl groups, enabling the nucleophilic addition reaction of ACCs to take place at higher potentials. Hence, ACCs are very suitable as electrode materials for LIBs. We have defined ACCs as a class of carbonyl compounds that can provide stable molecular structure and high activity carbonyl groups and have an aromatic ring structure. In terms of molecular structure, it is considered that p‐benzoquinones and 1,4‐dihydroxybenzene are isomers, so quinones are also classified as ACCs in this paper. **Table** **1** summarizes the classification of ACCs and the mechanisms of their reactions. According to the functional group, ACCs can be classified into four types.

1) Carboxylates. The carbonyl group is the active site of the electrochemical reaction. During the discharge process, two Li^+^ are embedded to form a newly conjugated system, and the carbonyl group is reduced to form an O—Li bond.
2) Imides and anhydrides. The carbonyl utilization ratio is 50% in order to maintain the relative stability of the heterocyclic structure of the molecule. The reversible transfer of two electrons, enhancement in the aromatic heterocyclic conjugated structure, and further dispersion of charge in the heterocyclic structure are favorable for the intercalation of Li^+^.
3) Ketones. Its active centers are two adjacent carbonyl groups, and the adjacent C—C bonds form double bonds after the loss of two electrons, which changes the structure into a stable enolated structure.
4) Benzoquinone. Benzoquinone is different from the above compounds with regard to the changes in the conjugated structure. The charge transfer reaction results in the formation of a new benzene ring, which improves the stability.

**Table 1:**
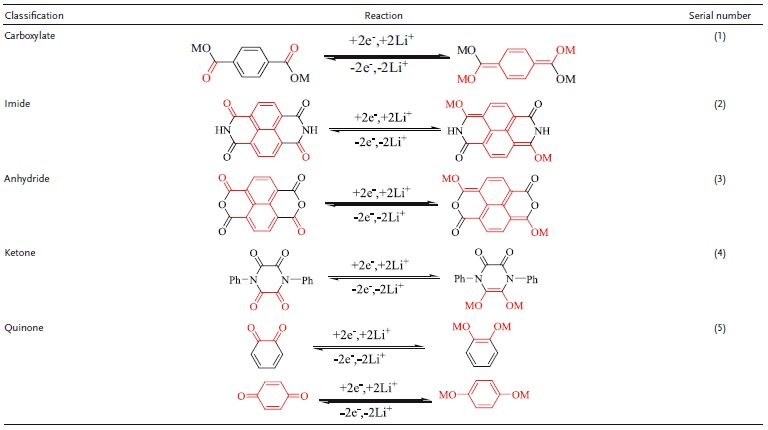
Reaction mechanism of aromatic carbonyl during charge–discharge

### 2.2 Coordination Theory of Polyvalent Cations

We chose lithium ion, having the smallest ionic radius, as the research target to exemplify the mechanism of the nucleophilic addition reaction. However, in the coordination theory of multivalent cations, the size of the ionic radius is not the only factor that affects the electrochemical reaction. It is also related to factors, such as the crystal structure of ions, the bond length between cations and organic molecules. Multivalent cations play two main roles in ACC materials. First, a stable coordination compound is formed by the chemical reaction of a multivalent cation and a CCC, and the stable organic layer and the inorganic layer are spaced apart to facilitate high‐speed, reversible storage and release of the cation; the coordination structure further enhances the molecular structure stability. The structure can stimulate additional aromatic rings as active centers, increase storage sites and increase capacity density.^59^, ^60^, ^61^, ^62^ Such a molecular structure is not only present in an organic solution battery, it also has great value in an aqueous solution battery. Second, the polyvalent cation can function not only as a fixed molecular structure but also as a stored cation to form a reversible coordination compound. Interestingly, the coordination compound formed by ACCs with polyvalent cations does not cause dissolution of the electrode with active materials, and this property can solve the dissolution loss due to the discharge product during the cycle.^63^, ^64^, ^65^ In aqueous batteries, the theory regarding the coordination of multivalent cations will become increasingly important.

## 3 Electrochemical Behavior of Aromatic Carbonyl Groups in Organic Electrolytes

### 3.1 Carboxylates

Carboxylates are a class of ACCs that containing polar groups. Equation (1) represents the nucleophilic addition reaction of lithium terephthalate. During the discharge, the carbonyl group of lithium terephthalate is reduced to form a dianion, which binds with the Li^+^ (or Na^+^, K^+^) in the electrolyte to form an O—Li bond, and the conjugated structure of the compound changes. Among the reactions, the degradation of the aromatic ring requires a large amount of energy, and the electrochemical reduction reaction takes place at a low potential.^66^ The *C*_theoretical_ of the simplest lithium terephthalate is 301 mAh g^−1^. Although the inorganic groups of carboxylates improve the polarity and ease the dissolution, the low rate performance and *V*_platform_ are still obstacles to the application of carboxylates. The electrochemical properties of all types of carboxylates with molecular structure modification are summarized in **Table** **2**.

**Table 2:**
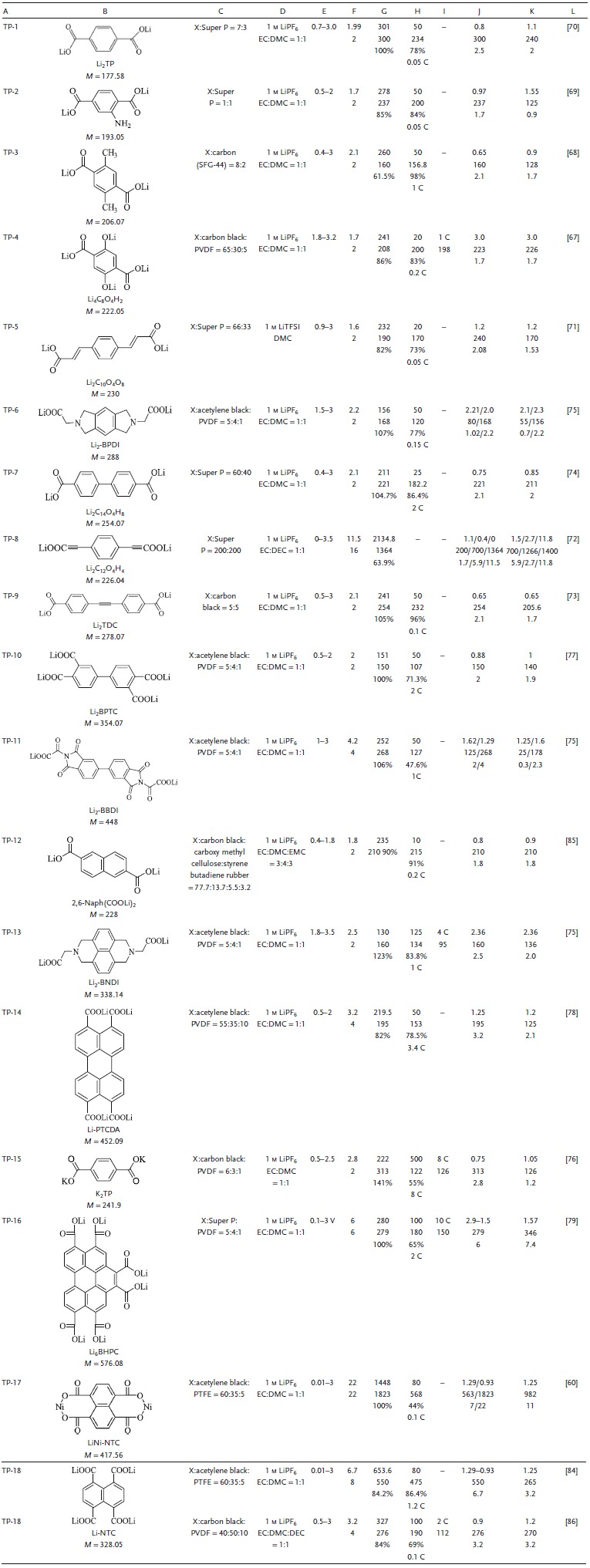
Summary of the electrochemical performance of representative carboxylate electrode materials in LIBs. A, Number. B, Structure formula, abbreviations, molecular weight (m, g mol^−1^). C, Electrode composition (X indicates active material, and the ratio is mass ratio.). D, Electrolyte. E, Voltage window. F, Experimental/theoretical mole number of electrochemical reaction. G, Theoretical capacity density, experimental capacity density, η. H, Number of cycles, maximum experimental capacity density (mAh g^−1^), capacity retention ratio, test rate. I, Maximum test ratio, capacity (mAh g^−1^). J, Voltage of discharge plateau (V), capacity density (mAh g^−1^), approximate number of reaction moles. K, Voltage of charge plateau (V), capacity density (mAh g^−1^), approximate number of reaction moles. L, Reference. TP: carboxylate; EC: ethylene carbonate; DEC: diethyl carbonate; DMC: dimethyl carbonate; DME: dimethoxyethane; DOL: dioxolame; EMC: ethyl methyl carbonate; TEGDME: tetraethylene glycol dimethyl ether; DMSO: dimethyl sulfoxide; PVDF: polyvinylidene fluoride; PTFE: polytetrafluoroethylene; LiTFSI: lithium bis(trifluoromethanesulfon) imide

#### 3.1.1 R‐Substituent Carboxylates

The R substituent increases the activity of the nucleophilic addition reaction by changing the electron cloud density of the carbonyl group in the organic molecular structure, thereby increasing the *V*_platform_. TP‐1, TP‐2, TP‐3, TP‐4, TP‐5, and TP‐6 represent the R substitutions of lithium salt terephthalate, such as those via salification,^67^ methyl groups,^68^ and amino groups.^69^ TP‐1 could deliver a reversible capacity of 300 mAh g^−1^, with a *V*_platform_ less than 1 V (0.8 V).^70^ Obviously, the nucleophilic addition reaction of TP‐1 was difficult to carry out. The capacity of TP‐2 with the introduced electron donor group was 237 mAh g^−1^ (*C*_theoretical_ is 278 mAh g^−1^), the capacity after 50 cycles was 200 mAh g^−1^, and the *V*_platform_ was 0.97 V. Since the electron withdrawing effect of the amino group N and the unshared p‐electron pair were delocalized by the conjugation effect and benzene ring, the electron cloud density of the benzene ring increased and the intercalation of Li^+^ was promoted. TP‐3 showed an initial capacity density (*C*_initial_) of 160 mAh g^−1^ with a *V*_platform_ of 0.6 V, and 98% of the *C*_initial_ was maintained after 50 cycles. The impact of the methyl group, showing a weak electron donating group, on its electrochemical properties was relatively small.^68^ In contrast, due to salification and control of the molecular size of TP‐4 to improve performance, nanosized TP‐4 exhibited a higher *V*_platform_ (3.0 V), higher capacity (241 mAh g^−1^, 86% of *C*_theoretical_), and superior cycle life. During charge–discharge, TP‐4 reacted in two phases; therefore, it could lithium/delithium at higher voltages.^67^

#### 3.1.2 π‐Core Structure of Carboxylates

**Figure 2:**
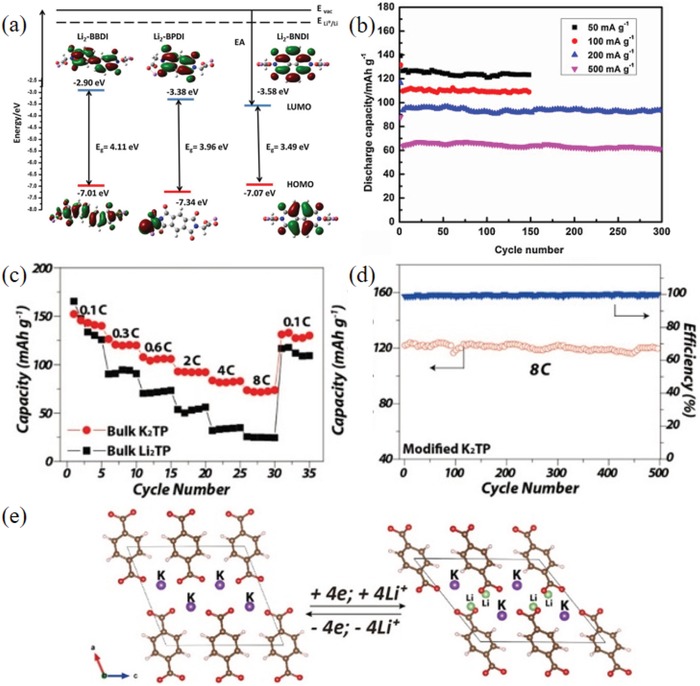
a) Energy level diagram and frontier orbitals of Li_2_‐BBDI (TP‐6), Li_2_‐BPDI (TP‐11), and Li_2_‐BNDI (TP‐13) generated from Gaussian 09 at the B3LYP/Lanl2DZ level. b) Cycling performance of the Li_2_‐BNDI at various current densities cycled between 1.8 and 3.5 V (vs Li/Li^+^).^75^ (Copyright 2016, Elsevier). c) Reversible redox mechanism for organic terephthalate (C_8_H_4_O_4_) motif. d) The initial three charge–discharge curves of K_2_TP. e) The selected profile at 8 C for 500 cycles. Reproduced with permission^76^ Copyright 2016, Elsevier.

The π structure is a general term for an additional conjugated structure of an aromatic ring core in ACCs, such as a benzene ring, an aromatic hetero ring, a double bond, a triple bond, etc. The π structure facilitates the averaging of electron clouds to reduce the LUMO of the molecule and promotes the nucleophilic addition reaction of the carboxylate. TP‐5 showed a *C*_initial_ of 190 mAh g^−1^ at 0.05 C, 73% of which was maintained after 20 cycles.^71^ It was apparent that the chain‐like conjugated double bond structure was not sufficiently stable during charge–discharge. The introduction of a triple bond could effectively improve molecular stability, for instance, of TP‐8 and TP‐9. TP‐8 exhibited a capacity of 200 mAh g^−1^ above 1 V and 1364 mAh g^−1^ below 1 V. This ultrahigh capacity was derived from the addition reaction of three bonds and the irreversible molecular structure.^72^ The three inactive bonds of TP‐9 increased the degree of electron cloud averaging of the π‐conjugated structure.^73^ TP‐9 delivered a high specific capacity of 254 mAh g^−1^ at 0.1 C, and retained 96% of its *C*_initial_ after 50 cycles at 100 C. The enhancement in the π‐conjugated structure of TP‐7 could reduce the interlayer spacing and improve the ionic conductivity. The capacity was 221 mAh g^−1^ at a low current density and was maintained 86.4% after 25 cycles at 2 C.^74^ Admittedly, the difference in the π structure also affected the interlayer spacing, which affects the diffusion rate of lithium ions, thus impacting the capacity density and rate performance. Comparing TP‐6, TP‐11, and TP‐13, the *V*_platform_ range of the heterocycle was shown to be high (1.62–2.36 V). With a change in the conjugated structure, the TP‐6, TP‐11, and TP‐13 interlayer spacing (5.29–10.30 Å) and free volume increased, which is favorable for Li^+^ storage. Corresponding to the highest occupied molecular orbital (HOMO) and LUMO levels of TP‐6, TP‐11, and TP‐13, their band gap (*E*_g_) values were 4.11, 3.96, and 3.49 eV, respectively (**Figure** **2**a). A lower energy of the LUMO resulted in higher electron affinity, which led to higher reduction potential, as shown in Figure 2a.^75^ The free volume of TP‐6, TP‐11, and TP‐13 increased with an increase in the layer spacing (5.29–10.30 Å), which was beneficial for Li^+^ storage. TP‐13, with a *V*_platform_ of 2.36 V and a *C*_initial_ of 160 mAh g^−1^, which is close to *C*_theoretical_, delivered charge capacities of 134 and 95 mAh g^−1^ after 125 cycles at 0.5 C and 300 cycles at 4 C, respectively (Figure 2b).

#### 3.1.3 Multiple Active Site Carboxylates

In the nucleophilic addition reaction of ACCs, the carbonyl involved in the reaction was called an efficient active site and increases the capacity density. The *V*_platform_ values of TP‐1 and TP‐10 were 0.8 and 1.29 V, respectively, and the capacity of TP‐10 after 50 cycles at 2 C was 107 mAh g^−1^.^77^ TP‐14 rendered an average operation voltage of ≈1.25 V and a high *C*_initial_ of 195 mAh g^−1^ with a stable cycle performance; the capacity after 50 cycles at 3.4 C was maintained 78.5%.^78^ TP‐16 synthesized by a facile Diels–Alder reaction not only stored nearly six electrons per molecule at 280 mA g^−1^ but also exhibited a good stable cycling performance over 100 cycles.^79^

#### 3.1.4 Other Cationic Carboxylates

**Figure 3:**
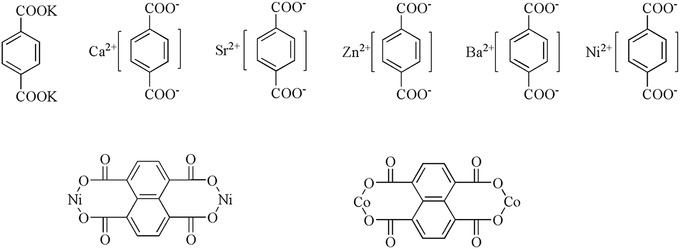
Structure of other cationic carboxylates.

In addition to lithium carboxylate, other cationic carboxylates can also be used as electrode materials (**Figure** **3**), e.g., those of K^+^,^80^ Na^+^,^81^ Ag^2+^,^82^ Co^2+^,^60^ Ca^2+^,^83^ Zn^2+^,^59^ Ni^2+^,^84^ Ba^2+^, and Sr^2+^.^61^ The lithium salt has the smallest radius, the highest electronegativity, and the smallest space for cation storage.

As shown in Figure 2c, PT‐16, with a good planar molecular structure, undergoes a four‐electron reaction in which Li^+^ replaces K^+^ during charge–discharge. The *C*_initial_ is up to 100% (313 mAh g^−1^), and the capacity is 55% after 500 cycles at 8 C (Figure 2d,e). The larger ionic radius due to K^+^ increased the stability of the lattice structure. K^+^ was electrochemically inert in K_2_TP, and the K—O bond was more covalent than the Li—O bond; hence, it could better inhibit dissolution in the electrolyte.^76^

Carboxylate molecules containing divalent cations mainly exhibit amorphous, crystalline, and hydrated states. The amorphous system exhibited an increase in the diffusion of Li^+^ and a shorter diffusion path compared with the three crystalline states of Zn^2+^.^59^ The capacity over 100 cycles at 0.5 C was 66% of *C*_initial_. The arrangement of alkali divalent metal cations in carboxylates has been investigated. Different from the Zn and two O‐suspension simple connections, the polyhedral Ca, Sr, and Ba are connected with a number of terephthalic acid esters to form a stable structure supported by an inorganic layer as an organic skeleton. Among them, the organic layer of Ca^2+^ exhibited the smallest distance, the shortest diffusion distance of Li^+^, and the largest diffusion coefficient.^61^ Although the electrochemical properties of carboxylates as electrode materials are generic, the inorganic nature of carboxylates can alleviate dissolution, and they can serve as substituents for other ACC molecular structures. Furthermore, the framework of different connection modes of different metal salts can improve the structural stability and electrochemical performance.

In summary, the conjugated structure in carboxylates was the determining factor that increased *V*_platform_, and the performances of the biphenyl and diphenyl were the most prominent. Additionally, the heterocyclic ring and the oxygen salt structure can further increase *V*_platform_, and the N heterocyclic ring and the oxy lithium salt can increase *V*_platform_ to greater than 1.5 V, which solves the low reaction potential of carboxylates. The number of active sites and other metal salt structures can increase the capacity, but the potential changes are minimal. With the exception of carboxylates with a high content of active sites (TP‐4), other carboxylates are not suitable for further research and production. The future research of carboxylates will tend to increase the content of active sites and dissolution inertia to achieve high storage and a longer life. However, the low discharge platform will be a fatal weakness, but this does not affect the carboxylate as a substituent to alleviate dissolution in organic electrolytes.

### 3.2 Aromatic Imides

Different from the low nucleophilic reactivity of carboxylates, the conjugated structure of aromatic imides is relatively stable, and the activity of the nucleophilic addition reaction is higher, increasing the *V*_platform_ to greater than 1.5 V. Equation (2) shows the reaction mechanism of a polyimide as an electrode material of an LIB and the number of electrons of the imide charge transfer reaction needed to avoid the formation of unstable double radicals. The discharge process forms radical anions and a dianion. Lithium enolate forms due to a conjugated stable structure of core p electrons.^47^, ^87^, ^88^, ^89^, ^90^, ^91^ Therefore, only 50% of the carbonyl groups in the aromatic imide participate in the nucleophilic addition reaction.^88^ The separation of aromatic imides into monomers and polymers based on two‐electron reactions is discussed.

#### 3.2.1 Monomeric Aromatic Imides

Solubility problems are a major challenge for monomeric imides as electrode active materials in LIBs. Modification of the molecular structure orientation can inhibit solubility and increase capacity, but the inhibition of solubility via molecular modification generally decreases the *C*_theoretical_. This section summarizes the solubility and the capacity density due to the aromatic π structure, substituted structure, and macromolecular structure, and the best optimization strategy is indicated. The electrochemical properties of all types of aromatic imide with molecular structure modification are summarized in **Table** **3**.

**Table 3:**
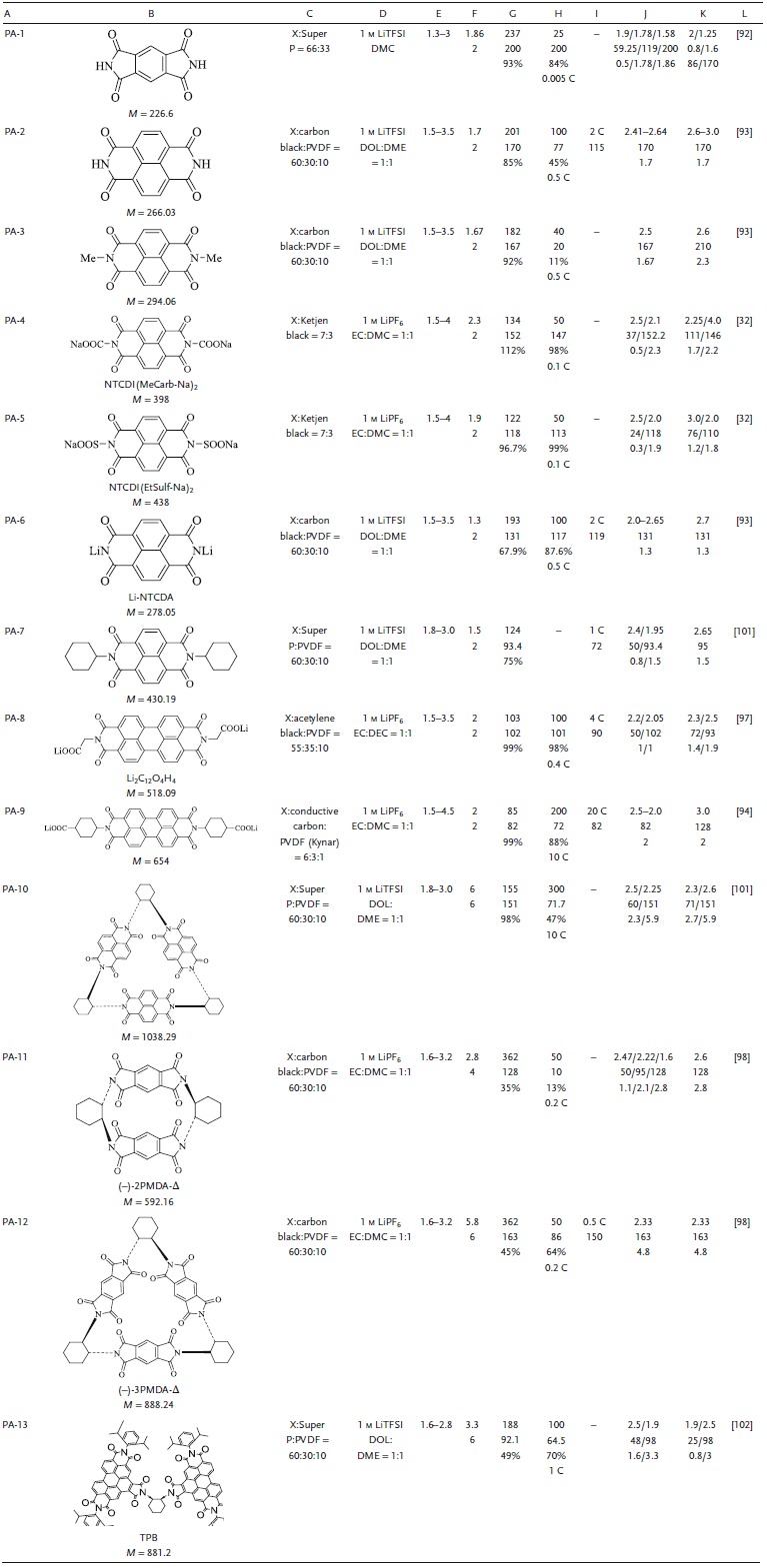
Summary of the electrochemical performance of representative aromatic imide electrode materials in LIBs. A, Number. B, Structure formula, abbreviations, molecular weight (*M*, g mol^−1^). C, Electrode composition (X indicates active material, and the ratio is mass ratio). D, Electrolyte. E, Voltage window. F, Experimental/theoretical mole number of electrochemical reaction. G, Theoretical capacity density, experimental capacity density, η. H, Number of cycles, maximum experimental capacity density (mAh g^−1^), capacity retention ratio, test rate. I, Maximum test ratio, capacity (mAh g^−1^). J, Voltage of discharge plateau (V), capacity density (mAh g^−1^), approximate number of reaction moles. K, Voltage of charge plateau (V), capacity density (mAh g^−1^), approximate number of reaction moles. L, Reference. PA: aromatic imide; EC: ethylene carbonate; DEC: diethyl carbonate; DMC: dimethyl carbonate; DME: dimethoxyethane; DOL: dioxolame; EMC: ethyl methyl carbonate; TEGDME: tetraethylene glycol dimethyl ether; DMSO: dimethyl sulfoxide; PVDF; polyvinylidene fluoride; PTFE: polytetrafluoroethylene; LiTFSI: lithium bis(trifluoromethanesulfon) imide

π‐*Core Structure of Monomeric Aromatic Imides*: PA‐1 (PMDA), PA‐2 (NTCDA), and PA‐3 (PTCDA) have different π conjugate structures.^87^, ^88^, ^91^ A larger π conjugate structure would have better stability. PTCDA, with a single‐electron reaction, exhibited good reversibility and cyclic stability in the potential range of 2.4–4.6 V. PTCDA delivered a *C*_initial_ of 131 mAh g^−1^ at 1 C, and 148 mAh g^−1^ capacity after 250 cycles; it also exhibited excellent cycle stability under the single‐electron reaction^91^ compared with the capacity contribution. PA‐1 was first embedded with 1.86 Li^+^ (220 mAh g^−1^); after over 200 cycles, the capacity retention was 84% (0.005 C).^92^ The carbonyl utilization efficiency of PA‐2 was 85% (*C*_theoretical_ is 201 mAh g^−1^), and after over 100 cycles, the capacity retention was 45% (0.5 C). The capacity at a high rate (2 C) was 115 mAh g^−1^.^93^ The difference in π structure highlighted the contradiction between capacity and cycle stability and showed that naphthalene with a π structure was best optimized.

**Figure 4:**
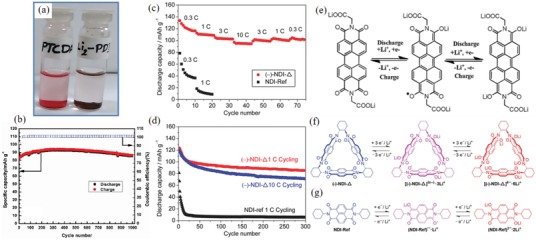
a) Solubility testing of PTCDA and Li_2_‐PDI (10 h standing after sonication) in a solution (4 mg mL^−1^) of 1:1 (v/v) EC:DMC. b) Long‐term cycling performance and coulombic efficiency of Li_2_‐PDI at 200 mA g^−1^ in the voltage window 1.5–3.5 V (vs Li/Li^+^). Reproduced with permission^97^ Copyright 2016, Elsevier. c) Cycling performance of the (−)‐NDI‐Δ and NDI‐Ref batteries, short‐term cycling performance of the (−)‐NDI‐Δ and NDI‐Ref batteries at different current rates. d) Long‐term cycling performance of the (−)‐NDI‐Δ and NDI‐Ref batteries at either a current rate of 1 or 10 C, showing good rechargeability of the (−)‐NDI‐Δ battery over 300 cycles compared with that of the NDI‐Ref battery. e) Schematic diagrams for the proposed electrochemical reactions during sodiation/desodiation of Li_2_‐PDI. Structural formulas and redox processes for (−)‐NDI‐Δ and NDI‐Ref. f) Redox reactions between (−)‐NDI‐Δ, [(−)‐NDI‐Δ] ^3(•−)^ · 3Li ^+^, and [(−)‐NDI‐Δ] ^6−^ · 6Li ^+^. Redox reactions between NDI‐Ref, (NDI‐Ref) ^•−^ · Li^+^, and (NDI‐Ref) ^2−^ · 2Li^+^. g) Each NDI unit is capable of undergoing two reversible one‐electron redox processes. Molecular triangles composed of three NDI units undergo two reversible three‐electron redox processes, amounting to a total of six electrons per molecule, whereas NDI‐Ref can only accept two electrons. Reproduced with permission^98^ Copyright 2015, Wiley‐VCH.

*R‐Substituent of Monomeric Aromatic Imides*: The R substitution structure changes the electrochemical performance due to the structural changes. Inorganic, strongly polarizing groups, such as lithiumoxy (O–Li), carboxylates, and sulfonates, inhibit dissolution. Small molecular groups, such as hydrogen, alkyl, and Li, affect *V*_platform_ by varying the electron cloud density of the carbonyl.^93^, ^94^, ^95^, ^96^ PA‐4 and PA‐5, containing —COONa (sodiumoxy carbonyl groups) and —SOOONa (sodium sulfonate groups), respectively, exhibited good solubility inertia.^32^ Their *C*_initial_ values were 134 and 122 mAh g^−1^, respectively, and their active materials utilization ratios were 100%. Both maintained a capacity retention of 99% after 100 cycles at 0.1 C. PA‐6, similar to PA‐4 and PA‐5, exhibited a *C*_initial_ of 131 mAh g^−1^, and its capacity after 100 cycles at 0.5 C was 87.6% of the *C*_initial_.^93^ **Figure** **4**a showed that PA‐8 with salt group had better dissolution inertia than PTCDA. When PTCDA was dissolved in EC: DMC, the supernatant turned red, indicating that PTCDA had greater solubility, while the supernatant of PA‐8 was still colorless, indicating that it was almost insoluble (Figure 4a). In terms of cyclic stability, it is worth noting that PA‐8 had the highest planarization of perylene and —CH_2_—COOLi structures. Hence, it exhibited excellent cycle life and rate performance. The *C*_initial_ was 102 mAh g^−1^, with a high *V*_platform_ and good reversibility. The —CH_2_—COOLi structure not only inhibited dissolution, but also enhanced the kinetics of the reaction through the super‐conjugated methylene ring. The *C*_initial_ at 4 C was 90 mAh g^−1^, and the capacity after 1000 cycles at 2 C was 87 mAh g^−1^ (Figure 4b).^97^

A large π‐structure combined with a salt structure could improve the cycle life and rate performance of ACCs. R‐substituted structures could reduce molecular energy and improve stability.^99^ Using perylene as the core structure, benzene substituted with nitrogen improves the electron affinity of the molecules during the discharge process. Carboxylic acids could alleviate the dissolution of active materials in organic electrolytes. Simultaneously, the N heterocyclic ring could also improve the electron mobility. The *C*_initial_ of PA‐9 at 10 C was 85 mAh g^−1^ (*C*_theoretical_ is 82 mAh g^−1^), and the capacity after 200 cycles was 72 mAh g^−1^. The capacity at a high rate of 20 C was 82 mAh g^−1^, which indicated fast kinetic performance and cycle stability.^94^ It was obvious that the structure of the salt and π–π aromatic ring structure of the monomeric imide were important for inhibiting dissolution and increasing *V*_platform_.

*3D Structure of Monomeric Imides*: 3D inert aromatic ring skeleton could effectively inhibit dissolution, and the microporous structure and rigid skeleton greatly improved the transportation of Li^+^ and the cycle life.^100^

As shown in Figure 4f, PA‐10, PA‐11, and PA‐12 have a cyclic or triangular 3D structure imide.^98^, ^101^ The rigid triangular structure and nanometer pore were not only structurally stable but also conducive to the diffusion of Li^+^. PA‐11 and PA‐12 exhibited spatial electron delocalization due to the forced proximity between the redox units. The triangular macrocyclic trimer was more stable than the dimer and the initial state of PMDI. PA‐12 showed a *C*_initial_ of 163 mAh g^−1^, indicating an acceptance of 4.8 Li^+^ per molecule, and retained 64% of the capacity after 50 cycles. In contrast, PA‐10 exhibited nearly perfect electrochemical performance (Figure 4c,d). This electrochemical stability and reversibility enables the sharing of electrons in a rigid triangular structure. The electron‐deficient orbital in (−)‐NDI‐Δ could be recycled permanently to form an intermediate ([(−)‐NDI‐Δ] ^3−^·3Li^+^ and [(−)‐NDI‐Δ] ^6−^·6Li^+^) of the stable charged reaction. PA‐10 showed a *C*_initial_ of 151 mAh g^−1^, indicating an acceptance of 5.9 Li^+^ per molecule. The capacity at 10 C after 300 cycles was 71 mAh g^−1^. Although the macromolecule of the 3D structure could effectively inhibit the dissolution, its increased relative molecular mass was obviously not conducive to an improvement in the energy/capacity density. However, the electrochemical properties of the 3D structure imide provided an idea for improving the conductivity of the imide.

The monomer imide has a large influence on the molecular structure due to the carbonyl structure, making the utilization ratio of the active carbonyl group only 50%, which greatly inhibits the increase of capacity density. However, the electrochemical properties of the polyimide, with multiple active sites, are also an indelible advantage. Insufficient capacity density of monomeric imides can increase the capacity density by constructing a molecular structure of multiple active sites and ensures solubility inertness and molecular structure stability. Clearly, the construction of 3D molecular structures and planar network molecular structures are effective measures.

#### 3.2.2 Aromatic Polyimides

Aromatic imides form aromatic polyimides via polycondensation reactions. The direct coupling between the aromatic imide monomers does not require any linkages, and polymerization enhances the conjugated structure to facilitate electron transport. Polymerization could effectively alleviate the solubility by ensuring that the average relative molecular mass was constant while increasing the total relative molecular mass. However, low conductivity was the main cause of low capacity density and poor rate performance.^103^ This part summarizes the molecular structure modifications and discusses effective measures for improving the inherent insulation, such as the use of mixed carbon materials. The electrochemical properties of the main polyimides are shown in **Table** **4**.

**Table 4:**
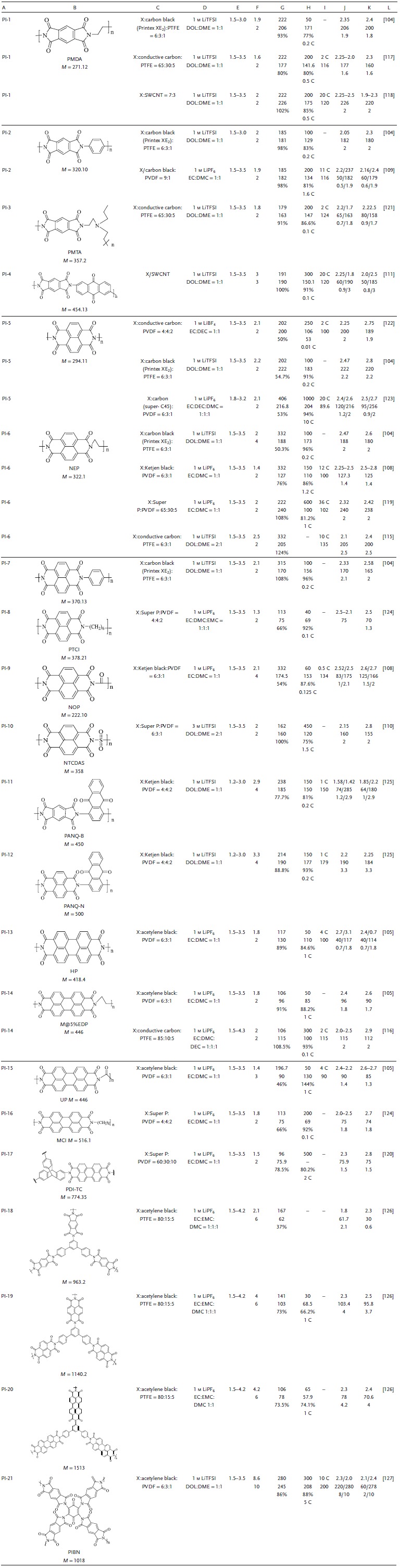
Summary of the electrochemical performance of representative polyimide electrode materials in LIBs. A, Number. B, Structure formula, abbreviations, molecular weight (*M*, g mol^−1^). C, Electrode composition (X indicates active material, and the ratio is mass ratio). D, Electrolyte. E, Voltage window. F, Experimental/theoretical mole number of electrochemical reaction. G, Theoretical capacity density, experimental capacity density, η. H, Number of cycles, maximum experimental capacity density (mAh g^−1^), capacity retention ratio, test rate. I, Maximum test ratio, capacity (mAh g^−1^). J, Voltage of discharge plateau (V), capacity density (mAh g^−1^), approximate number of reaction moles. K, Voltage of charge plateau (V), capacity density (mAh g^−1^), approximate number of reaction moles. L, Reference. PI: polyimide; EC: ethylene carbonate; DEC: diethyl carbonate; DMC: dimethyl carbonate; DME: dimethoxyethane; DOL: dioxolame; EMC: ethyl methyl carbonate; TEGDME: tetraethylene glycol dimethyl ether; DMSO: dimethyl sulfoxide; PVDF: polyvinylidene fluoride; PTFE: polytetrafluoroethylene; LiTFSI: lithium bis(trifluoromethanesulfon) imide

*π‐Core Structure of Aromatic Polyimides*: Benzene, naphthalene, and anthracene have different delocalized π‐bond stacking effects. A larger core π structure has a more average electron cloud distribution of chemical bonds, and the more stable structure would lead to a higher carbonyl activity and capacity density.^104^ PI‐1 exhibited a high *C*_initial_ of 206 mAh g^−1^ (1.9 Li^+^) and 77% capacity retention after 50 cycles at 0.2 C. PI‐6 rendered a high *C*_initial_ of 188 mAh g^−1^ (2 Li^+^), with stable cyclability (96% capacity retention after 50 cycles at 0.2 C).^105^ Polymers with three different π‐conjugated structures exhibited relatively good electrochemical properties. The larger π‐conjugated structure had a larger molecular structure, resulting in a decrease in capacity density, but the cycle stability and rate performance were improved. Due to the poor solubility and electron conductivity of the molecules with smaller π‐conjugated structures, high capacity and cycle stability could be achieved only at low current densities. Their rate performance could be improved by doping or modification of the molecular structure. Therefore, the molecular structures at different conjugate levels ultimately showed differences in electrochemical performance.

*R‐Substituent of Aromatic Polyimides*: The different R substituents in aromatic polyimides directly affect the molecular structure of aromatic polyimides. The substitution of alkyl and benzene rings can indirectly change the electron cloud density of carbonyl groups, and R structures with active carbonyl groups can increase the number of carbonyl groups participating in the reaction.^106^, ^107^ Substituted structures have chain,^108^ aromatic ring,^109^ and carbonyl structures.^108^ PI‐13 displayed a *C*_initial_ of 130 mAh g^−1^ and 94.7% capacity after 50 cycles at 1 C.^105^ The influence of substituents on the electrochemical properties was studied. The *C*_initial_ values of PI‐14 and PI‐15 were 115 and 90 mAh g^−1^, respectively. The average potential, energy density, power density, and cycle stability of PI‐14, PI‐13, and PI‐15 increased in turn. Similarly, the effects on the electrochemical properties of alkyl, carbonyl and benzene rings in R‐substituted structures with naphthalene and benzene as the core are consistent.^105^ PI‐10 exhibited an enhancement in the reduction potential to 2.15 V caused by the introduction of an S heteroatom and delivered a high specific capacity of 160 mAh g^−1^ and a long cycling stability, with 120 mAh g^−1^ after 450 cycles at 1.5 C.^110^

The benefit of the active sites was that they could increase the capacity, decrease the LUMO energy, and improve the electronic affinity of the molecules. PI‐4 displayed a superior cycling stability (191 mAh g^−1^ for the *C*_initial_, and 151 mAh g^−1^ after 300 cycles at 0.1 C) and a high rate capability (120 mAh g^−1^ at 20 C).^111^ Based on a comparison of the electrochemical properties of polyimides with different π structures and anthraquinone,^112^ PI‐11 and PI‐12 exhibited a high reversible capacity of 185 and 192 mAh g^−1^, respectively. After the 50th cycle, the capacity retentions were 81% and 93%, respectively.

Polyimides with additional active sites could effectively increase the capacity of the polymer. The appropriate number of active sites was an effective measure to solve the problem of excessive π structure and the decrease in capacity density.

*Composite‐Modified Polyimides with Carbon Materials*: The inherent insulation of polyimides could be improved by doping with high‐conductivity carbon materials. Small molecule carbon materials such as carbon black, SP, and KB can form dense networks, reduce pores, and increase the specific surface area for the reaction. Carbon‐based materials such as CNTs, multiwalled carbon nanotubes (MWCNTs), graphite, and carbon sheets can form a loose network structure with large pores, alleviating the volume change and realizing in situ polymerization. Single‐walled carbon nanotubes (SWCNTs) and graphene, with high specific surface areas, high electronic conductivity, and good mechanical properties, can increase the electron transport rate and toughness of electrode materials. The combination of CNT or graphene and polyimide to form a nanocomposite not only improved conductivity but also resulted in the formation of a π–π interaction with the connection between the conjugated aromatic rings.^113^ CNT and graphene were used as carriers, and polyimide was used as an active material. The π–π interaction resulted in the formation of a stable structure that could alleviate the solubility of polyimide and maintain a low capacity loss rate to improve cycle stability. Composites of SWCNTs with a network structure and imide rolling were used to fabricate electrode sheets suitable for bending.^114^ In situ polymerization of naphthalene tetracarboxylic dianhydride on the surface of functionalized graphene sheets (FGSs) yielded graphene nanocomposite PI‐6.^115^ Polyimide formed π–π bonds on the surface of graphene, which enhanced the intermolecular interaction and facilitated electron transfer. PI‐6 showed a reversible capacity of 205 mAh g^−1^ and a high rate capability (135 mAh g^−1^ at 10 C). The charge transfer resistance (*R*_ct_) of PI‐14 with CNTs composites was 265 Ω and that of pure PI‐14 was 1113 Ω, which showed that the electronic conductivity of composite CNTs greatly improved. They were stabilized via π–π interactions, resulting in a high reversible capacity of 106 mAh g^−1^ and a high capacity retention of 93% after 300 cycles, as well as 115 mAh g^−1^ at 2 C.^116^ The π–π action of the polyimide and carbon material improved the stability of the molecule, facilitated electron transport, and significantly improved the cycle stability and rate performance.

Pyromellitic dianhydride (PMDA) reversibly stored 1.9 Li^+^ (206 mAh g^−1^) and 77% capacity was retained after 50 cycles (155 mAh g^−1^ at 5 C). The inherent electrical insulation hindered the electron transport rate and resulted in an poor rate performance.^104^ After PI‐1 was combined with graphene, its *C*_initial_ was 177 mAh g^−1^, and the capacity was only 20% less after 200 cycles at 0.5 C.^117^ PI‐1 and SWCNTs were combined to prepare a flexible electrode, which had a porous structure to increase the contact area and improve electron transport. The *C*_initial_ was 226 mA g^−1^ and the capacity was 175 mAh g^−1^ after 200 cycles. SWCNTs also improved the cycle stability of PI‐1 and exhibited an excellent rate performance (120 mAh g^−1^ at 20 C).^118^ Different carbon materials have been combined with ACCs to significantly improve electrochemical performance.

**Figure 5:**
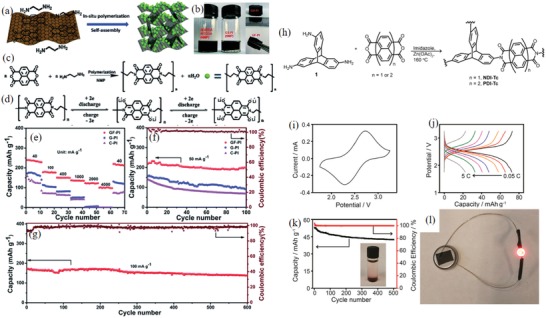
a) Schematic of the preparation process of GF‐PI. b) Photographs of precursor solution of GO, NTCDA and EDA, the resultant GF‐PI gel in NMP solution, the GH‐PI with interior NMP exchanged by water, and the final GF‐PI monolith after freeze‐drying and annealing. c) Synthetic route to PI. d) Electrochemical redox mechanism of PI for LIBs. Electrochemical characterization of GF‐PI as an LIB cathode. e) Rate performance of GF‐PI, G‐PI, and C‐PI at various current rates. f) Cycling performance of GF‐PI, G‐PI, and C‐PI at 50 mA g^−1^. g) Cycling performance of the GF‐PI electrode at 100 mA g^−1^. Reproduced with permission^119^ Copyright 2017, Royal Society of Chemistry. h) Synthesis of PDI‐Tc and NDI‐Tc frameworks. i) Cyclic voltammogram of the PDI‐Tc battery at 1 mV s^−1^. j) Galvanostatic charge/discharge curves of the PDI‐Tc battery from 0.05 to 5 C. k) Capacity decay and Coulombic efficiency of the PDI‐Tc battery over 500 cycles at 2 C. Inset: Photograph of PDI‐Tc in a 1 M LiPF_6_ ethylene carbonate dimethoxymethane (1:1 w/w) electrolyte. The light‐red color near the bottom is due to a suspension of solid PDI‐Tc near the sedimentation. l) A photograph of a red LED powered using a PDI‐Tc battery. Reproduced with permission^120^ Copyright 2017, American Chemical Society.

Graphene in situ polymerization of PI‐6 resulted in the formation of a 3D network structure (**Figure** **5**a–c).^119^ The *C*_initial_ was up to 240 mAh g^−1^ (Figure 5d), 40% of which was from the contribution of graphene. Compared with the reactivity of composite carbon, graphite, and graphene, GF‐PI‐6^119^ that was polymerized in situ in graphene exhibited the highest reactivity and a highly reversible redox peak of 2.3/2.42 V. GF‐PI‐6 also maintained a high capacity of 102 mAh g^−1^ at 4000 mA g^−1^ (Figure 5e). In contrast, the capacities of the other two carbon materials at 2000 mA g^−1^ were close to 0, and the capacity of GF‐PI‐6 was 120 mAh g^−1^. GF‐PI‐6, with low current density, maintained a capacity of up to 200 mAh g^−1^ (Figure 5f), and its capacity after 600 cycles at 1 C was 81.2% of the *C*_initial_ (Figure 5g). Summarizing the outstanding rate performance and excellent long cycle stability of GF‐PI‐6, the in situ polymerization of PI‐6, based on the 3D network structure of the graphene framework, promoted Li^+^ diffusion. It was further proved by electrochemical impedance spectroscopy (EIS) that the difference in *R*_ct_ before and after the 600th cycle was small (all less than 100 Ω). Therefore, the carbon material with an aromatic structure and the aromatic ring of the polyimide formed a π–π interaction that could improve the electronic conductivity and structural stability of the electrode material.

*3D Structure of Aromatic Polyimides*: Figure 5h shows the 3D backbone polymer of PI‐17, which reduced the solubility and improved the electronic conductivity, and the reaction formula of two imides and triptycene.^120^ The molecular structure shows that the imide with a larger conjugated core structure has more stable electrochemical properties. It showed a pair of reversible redox peaks of 2.3/2.7 V (Figure 5i) and a *C*_initial_ of 75.9 mAh g^−1^ at 2 C (Figure 5g), with a stable cyclability such that the capacity was maintained at 80.2% after 500 cycles (Figure 5k). Owing to the stability of the 3D skeleton of the molecule, the *R*_ct_ for EIS remained almost unchanged during the charge–discharge process (273.6–281.1 Ω), indicating that it has good kinetic stability. The future practicality of the imide electrode material is shown in Figure 5l.

#### 3.2.3 Conclusion

The solubility in organic electrolytes could be alleviated by introducing salt groups into the monomer imide molecule, and the optimum point of capacity density and solubility could be obtained for the imide with a suitable center conjugated aromatic ring naphthalene. Additionally, a rigid porous structure is beneficial for improving conductivity and dissolution inertia. Polymerization basically solved the problem of the active material dissolution. The introduction of an additional polymeric structure reduced the value of *C*_theoretical_. The aromatic monomer with active sites/polyimide was the means to increase the capacity density. The in situ polymerization with carbon‐based fiber polymers stabilized the charge transfer of +n or −n charge states by inducing the π‐conjugated structure to enhance the intermolecular interaction, resulting in a layer‐to‐layer molecular arrangement, which was conducive to ionic conductivity. To improve the magnification performance, the π‐conjugated structure could promote the dissolution inertia and improve the cycle stability. However, there are few studies on monomer imide and carbon‐based complexes. The main problem is that the connection between monomers and carbon‐based materials is not stable enough. Nevertheless, the superiority of carbon‐based materials and polymers will gradually attract researchers to build a stable bridge between monomers and carbon‐based materials. This will be a major breakthrough for small molecules to simultaneously solve the problem of conductivity and solubility.

### 3.3 Benzoquinones

Compared to imides, benzoquinone has a higher *V*_platform_ and a greater capacity density as an active material.^128^ Benzoquinone has two electrochemical principles (Equation (5)). On the one hand, during the discharge of *p*‐benzoquinone, the carbonyl group accepts electrons to form a second‐order anion, and a new benzene ring is formed in the six‐membered ring, which improves the molecular structure stability. To maintain the electrical neutrality principle, the second‐order anion forms O–Li with Li^+^ in the electrolyte. On the other hand, during the discharge of *o*‐benzoquinone, adjacent carbonyl groups form new double bonds and second‐order negative ions, which also form new benzene rings. However, the steric hindrance of benzoquinone is smaller, making it more likely to react; therefore, benzoquinone is the main research focus.

#### 3.3.1 Monomeric Benzoquinones

Monomeric benzoquinone has a high capacity density and high solubility, allowing it potential applications in liquid flow batteries but not in solid‐state batteries.^129^ The solubility of benzoquinone in electrolytes can be alleviated by modifying the molecular structure and loading insolubles; in this way, the electrochemical performance of benzoquinone can be improved. It is important to summarize the relationship between molecular structure and solubility for the prediction and design of insoluble benzoquinones. **Table** **5** summarizes the electrochemical properties of major benzoquinone molecules in recent years.

**Table 5:**
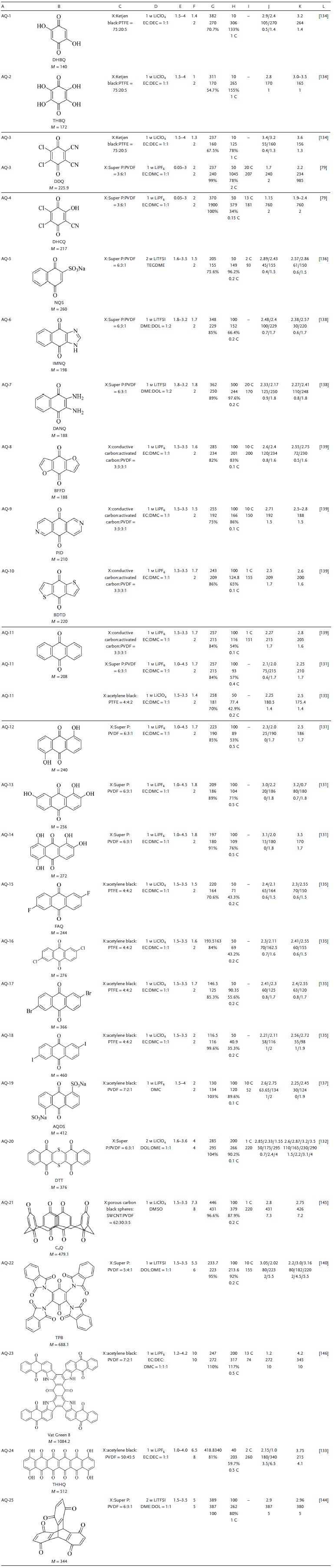
Summary of the electrochemical performance of representative quinone electrode materials in LIBs. A, Number. B, Structure formula, abbreviations, molecular weight (*M*, g mol^−1^). C, Electrode composition (X indicates active material, and the ratio is mass ratio). D, Electrolyte. E, Voltage window. F, Experimental/theoretical mole number of electrochemical reaction. G, Theoretical capacity density, experimental capacity density, η. H, Number of cycles, maximum experimental capacity density (mAh g^−1^), capacity retention ratio, test rate. I, Maximum test ratio, capacity (mAh g^−1^). J, Voltage of discharge plateau (V), capacity density (mAh g^−1^), approximate number of reaction moles. K, Voltage of charge plateau (V), capacity density (mAh g^−1^), approximate number of reaction moles. L, Reference. AQ: quinone; EC: ethylene carbonate; DEC: diethyl carbonate; DMC: dimethyl carbonate; DME: dimethoxyethane; DOL: dioxolame; EMC: ethyl methyl carbonate; TEGDME: tetraethylene glycol dimethyl ether; DMSO: dimethyl sulfoxide; PVDF: polyvinylidene fluoride; PTFE: polytetrafluoroethylene; LiTFSI: lithium bis(trifluoromethanesulfon) imide

*π‐Core Structure of Monomeric Benzoquinones*: An extra π structure can promote the intramolecular averaging of the electron cloud and increase the electrochemical reactivity of the carbonyl group, whereas the rigid benzene ring can reduce the solubility to some extent. The simplest high solubility of 1,4‐*p*‐benzoquinone has been mainly used in flow batteries to confirm the solubility limitations of monomeric benzoquinone.^130^ AQ‐12^131^ dissolved in an inert additional π structure reduced the *V*_platform_ (2.3 V). The utilization of AQ‐12 was 84%, and it still exhibited a capacity retention of 57% after 100 cycles. The results showed that the solubility was greatly alleviated and the *V*_platform_ could be increased by increasing the number of active sites. The carbonyl utilization ratio of AQ‐20 was up to 100%, and the *V*_platform_ was 2.59 V, which indicated that the highly active sites promoted the nucleophilic addition of the carbonyl group.^132^ However, because of the narrow spatial structure, the carbonyl group activity decreased. The utilization of AQ‐24‐carbonyl was 81% (418.8 mAh g^−1^ of *C*_theoretical_), and the capacity after 200 cycles at 0.1 C was only 59.6% of the *C*_theoretical_.^133^ Therefore, the magnitude of carbonyl activity directly affects the capacity density.

**Figure 6:**
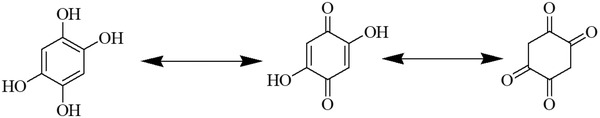
Isomer of benzoquinone.

*Substitution Reaction of Monomeric Benzoquinones*: The size of the conjugate structure can only determine the *C*_initial_, and the cycle stability is also affected by substituents. In benzoquinone, hydroxyl substitution is considered to result in an inactive carbonyl isomer (**Figure** **6**) and can improve the reactivity of the carbonyl group. AQ‐1 and AQ‐2 had high *V*_platform_ values of 2.9 and 2.8 V, respectively. The capacity of AQ‐1 was 270 mAh g^−1^.^134^ The dissolution of AQ‐1 and AQ‐2 was alleviated by quasi‐solid‐state and ionic liquid electrolytes, resulting in a high capacity retention of 100% after 10 cycles at 1 C. As the number of hydroxyl groups increased, the utilization of the carbonyls of AQ‐11, AQ‐12, AQ‐13, and AQ‐14 increased (≈70–91%), the *V*_platform_ increased (2.25–3.1 V), as did the cycle stability in the 100th cycle at 0.5 C.^131^ AQ‐14 exhibited a high reversible capacity of 180 mAh g^−1^ (*C*_theoretical_ is 197 mAh g^−1^), a *V*_platform_ as high as 3.1 V, and a stable cyclability with 109 mAh g^−1^ capacity retention over 100 cycles at 0.5 C. In summary, the addition of an appropriate hydroxyl group to benzoquinone could increase the carbonyl utilization rate and increase the *V*_platform_. The electron cloud density of the benzene ring was reduced after the introduction of the electron withdrawing group, which was favorable for the formation and stabilization of the *n*‐order anion, thereby promoting the nucleophilic addition reaction of the carbonyl group. A halogen changed the electron cloud density of AQ‐15, AQ‐16, AQ‐17, and AQ‐18 via the induction effect, thereby increasing the nucleophilic addition reaction, which was beneficial for increasing the *V*_platform_.^135^

The *V*_platform_ of AQ‐15 was 2.4 V. The utilization of the carbonyl in AQ‐18 was 99.6%. AQ‐17 has the most outstanding cyclic stability, and its capacity after 50 cycles at 0.2 C was 55.6% of the *C*_theoretical_. The *C*_theoretical_ is determined using the density and molar volume. Therefore, the capacity of iodine substitution would be closest to the *C*_theoretical_. However, the relative molecular mass of AQ‐18 limited the capacity. Moreover, the electron withdrawing effect of the halogen was not strong; hence, the halogen substitution resulted in a 50th cycle capacity retention of less than 55.6% at 0.2 C.

**Figure 7:**
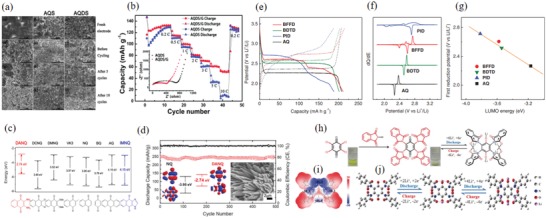
a) SEM of AQ (AQ‐11), AQS (AQ‐5), and AQDS (AQ‐19) electrodes: a–c) fresh electrode, d–f) before cycling, g–f) after 5 cycles, and j–l) after 10 cycles. b) Rate performance of AQDS and AQDS/G paper electrodes. The inset figure shows the Nyquist plots of AQDS and AQDS/G paper electrodes. Reproduced with permission^137^ Copyright 2014, Royal Society of Chemistry. c) HOMO/LUMO energy diagrams and morphologies of the DANQ and IMNQ compared to those of various NQ‐derivatives. The HOMO–LUMO gap is indicated. d) Discharge/charge capacities and coulombic efficiencies of the Li‐DANQ cell at 0.2 C with an extended life of 500 cycles. Reproduced with permission^138^ Copyright 2016, American Chemical Society. e) Discharge–charge profiles of the cells at 0.1 C at the second cycle. f) Differential capacity curves derived from the discharge‐charge curves shown in (e). g) Correlation between the first reduction potentials and the calculated LUMO energies. Reproduced with permission^139^ Copyright 2013, Wiley‐VCH. h) The preparation and reversible electrochemical redox mechanism of TPB/Li_6_TPB (molar mass of TPB is 689 g mol^−1^, *C*_theoretical_ is 233 mAh g^−1^). The digital photos show the high solubility of the raw materials and the insoluble character of TPB. i) The ESP (kcal mol^−1^) mapped molecular vdW surface of the TPB molecule. j) Configurations of TPB/Li_2_TPB/Li_6_TPB. Reproduced with permission^140^ Copyright 2017, Wiley‐VCH.

Distinctly, the sodiumoxy carbonyl group monomer in substituted benzoquinone had a stronger electron withdrawing effect than the halogen.^99^ The *V*_platform_ of AQ‐5 was raised to 2.89 V, and the capacities at 0.2 C after the 1st and 50th cycles were 155 and 149 mAh g^−1^, respectively; a capacity of 92 mAh g^−1^ was maintained several times under 2 C.^136^ Further study of the electron withdrawing effect of sodium showed that the *C*_initial_ of AQ‐19 was up to 100% (134 mAh g^−1^) and its polarity was suppressed, improving the solubility of sodium in the electrolyte; the capacity after 100 cycles at low current density was maintained at 90% of the *C*_initial_.^137^ The change in morphology of the molecules of different sodium sulfonate groups during charge–discharge showed that the introduction of two sodiumoxy carbonyl groups resulted in good solubility inertness (**Figure** **7**a). The introduction of graphene improved the electron conductivity and accelerated the electron transport rate, which was 52 mAh g^−1^ at 10 C (Figure 7b). However, sodiumoxy carbonyl groups (—SO_3_Na), with a higher molecular weight, reduced the *C*_theoretical_ of the monomer benzoquinone. Two amino substitutions increased the *V*_platform_ of AQ‐7.^138^ AQ‐6 and AQ‐7 delivered *V*_platform_ values of 2.48 and 2.33 V, respectively. AQ‐7 exhibited a high capacity retention of 97.6% at 0.2 C after 500 cycles and a high capacity of 170 mAh g^−1^ at 20 C (Figure 7d). The hydroxyl group (OH), sodiumoxy carbonyl groups (SO_3_Na), and amino group (NH_2_) in the substitution structure clearly had a good effect by relieving dissolution and increasing the *V*_platform_.

*Monomeric Quinone Heterocyclic Structure*: The influence of substituent groups on the electrochemical properties resulted from the altered electron cloud density and molecular polarity. These changes were fundamentally related to the introduction of heteroatoms and reflect the effect of heteroatoms on the electrochemical properties of benzoquinone monomer.

The main elements of the heterocyclic ring in the quinone molecule were O, N, and S, with an electronegativity greater than that of C.^141^ The role of the O, N, and S in the heterocyclic structure in the electrochemical performance of AQ‐8, AQ‐9, and AQ‐10 was investigated. It was clearly seen that the *V*_platform_ corresponding to the first stage of the redox reaction increased in the order of AQ‐11 (AQ, 2.27 V), AQ‐10 (BDTD, 2.52 V), AQ‐8 (BFFD, 2.61 V), and AQ‐9 (PID, 2.71 V) (Figure 7h).^139^ The carbonyl utilization of different heterocyclic benzoquinone molecules increased to 82–88% (Figure 7i), and the *C*_initial_ of AQ‐8, AQ‐9, and AQ‐10 were as high as 208–234 mAh g^−1^. The capacities of AQ‐8, AQ‐9, and AQ‐10 after 100 cycles were 54–86% of the *C*_initial_, and it was apparent that the heterocyclic structure could promote the nucleophilic addition reaction. The relationship between the *V*_platform_ and the LUMO energy showed that the smaller the LUMO, the higher the electron affinity and the higher the *V*_platform_ (Figure 7j). Therefore, AQ‐9 exhibited the best electrochemical performance. The electron conductivity further improved due to graphene modification, and a high capacity of 150 mAh g^−1^ was obtained at 10 C.

Heterocyclic structures increased the electrochemical activity of carbonyl groups due to the inclusion of larger electronegative atoms, but the additional inactive structures reduced the *C*_theoretical_.^142^, ^143^ AQ‐22 presented a heterocyclic structure with an active site that increased the high *V*_platform_ (3.05 V), and the number of active sites increased to as high as 10. Simultaneously, the rigid skeleton structure rendered the solubility of AQ‐22 extremely low, and the formation of a new conjugated π system reduced the LUMO energy level.^140^ The reaction mechanism and dissolution inertness of AQ‐22 are shown in Figure 7k. Using the molecular model of electrostatic potential (ESP), it was proved that the top and bottom carbonyl reactions were the first in the embedding process, followed by those of the carbonyl groups on both sides (Figure 7l). The relative stability of the molecular structure is shown in Figure 7m. Even after 100 cycles, the capacity retention at 0.2 C was 91.4%, and the discharge capacity at 10 C was still 155 mAh g^−1^.

In summary, a structure containing an active site heterocyclic ring was more advantageous for improving electrochemical properties. Atoms with greater electronegativity could better attract electron clouds of monomeric benzoquinone molecules and increase nucleophilic addition reaction activity.

*3D Structure of Benzoquinones*: Although planar molecules could express good electrochemical properties, the 3D structure combined with the advantages of the former can further improve the molecular stability and carbonyl activity.

**Figure 8:**
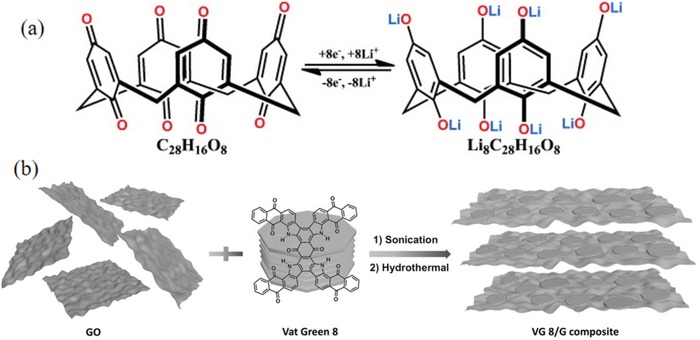
a) Molecular formula and proposed electrochemical redox reactions of C_28_H_16_O_8_ (C_4_Q) to give Li_8_C_28_H_16_O_8_; its *C*_theoretical_ is 446 mAh g^−1^. Reproduced with permission^145^ Copyright 2013, Wiley‐VCH. b) Schematic illustration of the synthesis of VG 8/G composite, which was formed through the disassembly of the Vat Green 8 crystal structure and subsequent reassembly on the graphene sheets. The main driving force for the formation of the VG 8/G hybrid architecture is the π–π interactions between Vat Green 8 and the graphene sheets. Reproduced with permission^146^ Copyright 2016, Wiley‐VCH.

The molecular engineering‐designed 3D structure exhibited good solubility inertia and high activity and electronic conductivity.^144^ As shown in **Figure** **8**a, AQ‐21, with eight active sites, exhibited a high capacity of up to 431 mAh g^−1^ (446 mAh g^−1^ of *C*_theoretical_).^145^ Even after 100 cycles at 0.2 C, the capacity was 379 mAh g^−1^. In addition, when the current was elevated to 1 C, the discharge capacity was maintained at 220 mAh g^−1^. The formation of a 3D structure with a low steric hindrance and a highly active site via a short‐chain linkage could promote the transport of Li^+^ and inhibit dissolution. A composite material with a 3D structure was synthesized by combining benzoquinone with a carbon material. The electrostatic interaction between AQ‐23 and the graphite layer yielded a 3D structural model (Figure 8b) that promoted the diffusion of Li^+^. The graphite layer acted as a current collector to accelerate the movement of electrons. The capacities for the initial and 200th cycles of AQ‐23 at 0.5 C were 272 and 317 mAh g^−1^, respectively, and it still maintained a discharge capacity of 74 mAh g^−1^ even at 13 C.^146^

In organic electrolyte, the solubility of benzoquinone is the best among similar ACC materials, but the carbonyl activity of benzoquinone is also the highest among similar ACC materials. Among the monomeric benzoquinones, the benzoquinone with a high volume density and a high release point platform is one of the most promising materials, but its solubility is a fatal weakness hindering its development. The introduction of polar substituents and heterocyclic rings into the molecular structure can partially alleviate the dissolution but will reduce the capacity density. Attaching it to a conductive carbon material can maintain its electrochemical properties, but costs more. In our opinion, a reasonably designed heterocyclic aromatic structure and monomer benzoquinone with multiple active sites would effectively be able to alleviate the dissolution, such as a dimer, trimer, and 3D structure, as well as a more economical insoluble carrier, such as SiO_2_.

#### 3.3.2 Polybenzoquinones

Monomer benzoquinones undergo polycondensation to form a polymer, which can effectively improve the solubility inertia of the electrode active material. The sulfur atom and —OH in the monomer benzoquinone could increase the *V*_platform_, enhance the electrochemical activity, and promote the binding/release of Li.^58^, ^147^ The characteristics of polybenzoquinone with a sulfur atom or a hydroxyl group in the molecular structure are subsequently summarized. For the electrochemical activity of the carbonyl, the influence of the degree of polymerization of the polymer, steric hindrance of the polymer, and number of substitutions are given. A summary of the electrochemical properties of the main polybenzoquinone molecules are shown in **Table** **6**.

**Table 6:**
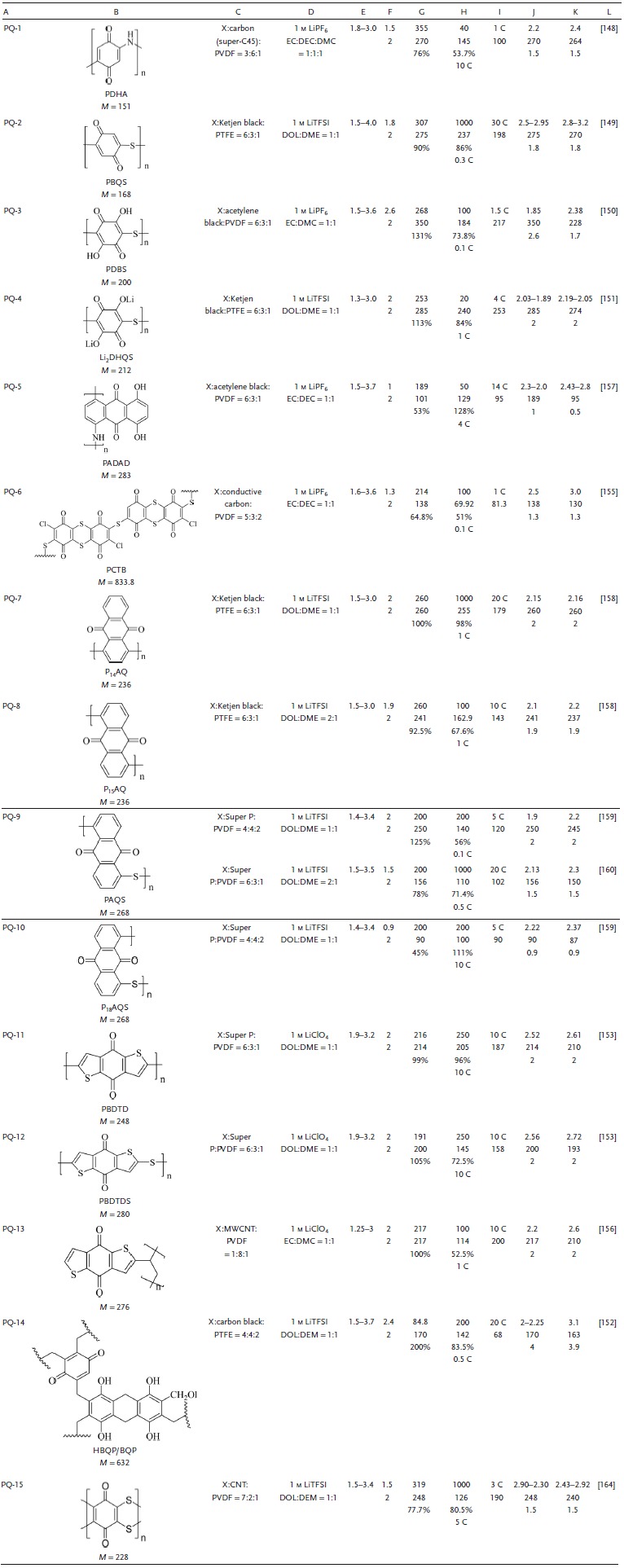
Summary of the electrochemical performance of representative polybenzoquinone electrode materials in LIBs. A, Number. B, Structure formula, abbreviations, molecular weight (*M*, g mol^−1^). C, Electrode composition (X indicates active material, and the ratio is mass ratio). D, Electrolyte. E, Voltage window. F, Experimental/theoretical mole number of electrochemical reaction. G, Theoretical capacity density, experimental capacity density, η. H, Number of cycles, maximum experimental capacity density (mAh g^−1^), capacity retention ratio, test rate. I, Maximum test ratio, capacity (mAh g^−1^). J, Voltage of discharge plateau (V), capacity density (mAh g^−1^), approximate number of reaction moles. K, Voltage of charge plateau (V), capacity density (mAh g^−1^), approximate number of reaction moles. L, Reference. PQ: poly‐benzoquinone; EC: ethylene carbonate; DEC: diethyl carbonate; DMC: dimethyl carbonate; DME: dimethoxyethane; DOL: dioxolame; EMC: ethyl methyl carbonate; TEGDME: tetraethylene glycol dimethyl ether; DMSO: dimethyl sulfoxide; PVDF: polyvinylidene fluoride; PTFE: polytetrafluoroethylene; LiTFSI: lithium bis(trifluoromethanesulfon) imide

**Figure 9:**
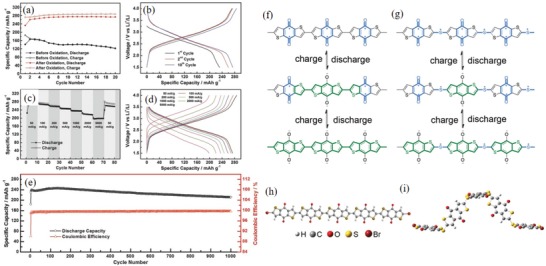
a) Discharge/charge capacity profiles versus cycle number under 50 mA g^−1^ for PBQS samples before and after oxidation. b) Voltage profiles of the 1st, 2nd, and 10th cycle for PBQS after oxidation. c) Discharge/charge capacity profiles versus cycle number under sequentially changed current rates from 50 to 5000 mA g^−1^. d) Corresponding voltage profiles under different current rates. e) Long‐term cycling profiles within 1000 cycles under 500 mA g^−1^. Reproduced with permission^149^ Copyright 2015, Wiley‐VCH. f–g) Conjugation evolution during the discharge/charge process. Asterisk (*) indicates the bifurcation point in the BDT unit and at S atoms. In PBDTD, cross conjugation (blue) evolves through conjugation (green) during lithiation. In PBDTDS, cross conjugation (blue) remains during lithiation due to the bifurcation points at S atoms. h–i) Optimized molecular conformations of PBDTD and PBDTDS using standard B3LYP/6‐31G (d, p) calculations. Planar conformation of Br(BDTD)_5_Br and helical conformation of Br(BDTDS)_4_BDTDBr. Reproduced with permission^153^ Copyright 2017, Elsevier.

*Substitution Reaction of Polybenzoquinones*: The *V*_platform_ of amino‐substituted PQ‐1 was 2.2 V, the initial and 40th cycle capacities at 0.1 C were 270 and 145 mAh g^−1^, and the capacity was 100 mAh g^−1^ at 1 C. These results show that the higher the degree of polymerization, the better the solubility and inertness, and the more stable the cycle performance of the battery.^148^ DFT calculations of PQ‐2 showed a lower LUMO energy level with a higher electron affinity and *V*_platform_. The complete oxidation of the hydroxyl group increased the number of electrochemically active sites; the capacity of the carbonyl groups is shown in **Figure** 9a. Its reversible capacity was 236.5 mAh g^−1^ (*C*_theoretical_ is 307 mAh g^−1^) (Figure 9b). At 100, 200, 500, 2000, and 5000 mA g^−1^, the capacities were respectively maintained at 98%, 94%, 86%, 80%, and 72% of the *C*_initial_ (Figure 9c,d), showing outstanding rate performance. The capacity was retained 86% after 1000 cycles (Figure 9e).^149^ PQ‐3 exhibited a capacity of 350 mAh g^−1^ (131% of the *C*_theoretical_), with a capacity retention of 74% at 1 C and a capacity of 217 mAh g^−1^ at 1.5 C.^150^ PQ‐4 exhibited a capacity retention of 84% after 20 cycles at 1 C. The high degree of polymerization and —S— resulted in a stable molecular framework and a highly reversible reaction.^151^ Hydroxyl‐substituted PQ‐14, an additional conjugated structure that enhanced the structural stability, exhibited a high capacity density. The initial and 200th cycle capacities at 0.5 C were 200% and 83.5% of the *C*_initial_, and the capacity was 68 mAh g^−1^ at 20 C. This indicated that the hydroxyl group substitution in polybenzoquinone hydrazine reduced the solubility of the polymer and improved the cycle stability, which was consistent with the result of the hydroxyl group substitution in the monomer quinones.^152^

*Polybenzoquinones with Heterocyclic Structure*: The monomer benzoquinone with sulfur heterocyclic structure exhibited outstanding electrochemical performance. The polybenzoquinones with S heterocyclic structure also showed good electrochemical properties.^154^ PQ‐6 had a high *V*_platform_ of 2.5 V and a capacity of 138 mAh g^−1^. The capacity after 100 cycles was retained at 51% (0.1 C) and was 81.3 mAh g^−1^ (1 C).^155^ PQ‐11 and PQ‐12 contained a thiophene heterocycle of the S element. The PQ‐12 (PBDTDS) molecular backbone was linked to the —S— bond.^153^ Figure 9f,g shows that the cross‐conjugated structure of the BDTD unit became a conjugated structure after PQ‐11 was completely reduced, and PQ‐12 contained —S— cross‐coupling of polymers at intersections; different discharge states led to different electronic conductivities and rate performances. It was calculated by DFT that Br(BDTD)_5_Br exhibited a stable planar structure (Figure 9h,i). The ordered π structure was conducive to electron transport. The presence of the C—S—C bond led to inefficient Br(BDTDS)_4_BDTDBr with π low key superimposed spiral structure. The capacity was 99% of the *C*_theoretical_ (200‐214 mAh g^−1^), and the capacities of PQ‐11 and PQ‐12 after 250 cycles at 0.1 C were 96% and 72.5%, respectively. Their capacities at 10 C were 187 and 158 mAh g^−1^, respectively. The electrochemical performance of PQ‐11 was consistent with the molecular structure results. This further illustrated the critical role of the structure for electrochemical performance. PQ‐13 presented a short chain, forming a flexible electrode, and the inactive extra structure reduced the capacity (217 mAh g^−1^) and redox potential (2.2 V). The capacity after 100 cycles was maintained at 52.5% of the *C*_initial_ (200 mAh g^−1^ at 10 C).^156^

**Figure 10:**
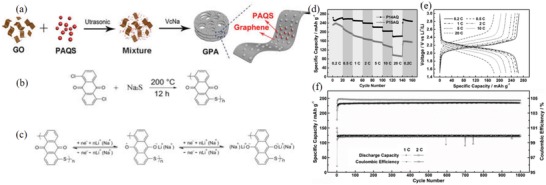
a) Schematic of the synthesis process of GPA. b) Synthetic route to PAQS. c) Reversible redox mechanisms of PAQS in LIBs and SIBs. Reproduced with permission^160^ Copyright 2017, American Chemical Society. d) Discharge capacity profiles versus cycle number for P14AQ and P15AQ at different current rates (from 0.2 to 20 C). e) Corresponding voltage profiles of P14AQ at different current rates. f) Long‐term cycling profiles (1000 cycles) of P14AQ at 1 and 2 C. Reproduced with permission^158^ Copyright 2015, Wiley‐VCH.

*Polymeric Structure Isomerized Polybenzoquinones*: The molecular structure stability of polybenzoquinone at different polymerization sites is very different, which directly leads to alterations in the electrochemical activity of the carbonyl and impacts the difficulty of the nucleophilic addition reaction. Ipsilateral‐polymerized PQ‐5 exhibited a *C*_initial_ of 101 mAh g^−1^, and the 50th cycle capacity was up to 128 mAh g^−1^, with a high rate performance (95 mAh g^−1^ at 7 C).^157^ The steric hindrance of PQ‐7 was smaller than that of PQ‐8.^158^ Figure 10e shows that the potential difference between the PQ‐7 charge and discharge was only 0.04 V, and the *V*_platform_ was also higher than that of PQ‐8 (2.1 V). The band gaps, by DFT calculations, of PQ‐7 and PQ‐8 were −2.65 and −2.63 eV, respectively, showing that PQ‐7 exhibited a small steric hindrance and high electron affinity. The *C*_initial_ of PQ‐7 and PQ‐8 were 260 and 241 mAh g^−1^, respectively, and PQ‐7 had a capacity of 179.4 mAh g^−1^ at 20 C. PQ‐8 exhibited only 143 mAh g^−1^ at 10 C (Figure 10e). The capacity retention of PQ‐7 after 1000 cycles at 1 C was 98% (*C*_theoretical_ was 255 mAh g^−1^) and that of PQ‐8 after 100 cycles was 67.6%, highlighting the significant long cycle stability of PQ‐7 (Figure 10f). The PQ‐10 steric hindrance for different side polymerizations was smaller than that of PQ‐9, and the carbonyl activity was higher.^159^ The electrochemical difference was due to the difference in the initial carbonyl utilization rates. The PQ‐9 and PQ‐10 carbonyl utilizations were 100% and 45%, respectively. The capacities of PQ‐9 and PQ‐10 after 200 cycles at 0.1 C were 140 and 100 mAh g^−1^, respectively, and PQ‐9 exhibited a capacity of 120 mAh g^−1^ at 5 C. The optimization of the polybenzoquinone isomer structure was an effective method to improve the structural stability of the polymer and promote the nucleophilic addition reaction.

*Composite Stereo Structure of Polybenzoquinones*: The in situ polymerization of graphene could establish a 3D structure, which effectively improved the solubility inertia and increased the ionic conductivity. The expression of the electrochemical properties of PQ‐9 was hampered due to its high solubility and large steric hindrance. The reaction mechanism of graphene‐encapsulated PQ‐10 composites ((graphene/poly (anthraquinonyl sulfide) (PAQS) composite aerogel (GPA)) was a two‐electron transfer. The electronic conductivity was enhanced by the π—π bond stacking of graphene and PQ‐9. The 3D porous structure provided efficient continuous channels and multidimensional electron transport paths for efficient ion transport, resulting in fast dynamics (**Figure** **10**a).^160^ The utilization of carbonyl increased to 78%, and the capacity retention after 1000 cycles at 0.5 C was as high as 71.4%, with a high capacity of 102 mAh g^−1^ at 20 C. The 3D structure formed by the GO composite PQ‐9 material improved its electrochemical properties.

The study of polybenzoquinone in LIBs is a way for monomer benzoquinone to address the solubility problem. Although polybenzoquinone can solve the problem of solubility, at the same time there are other problems. Polymerization has a steric hindrance effect between monomers, where the activity of active sites is affected as are the actual reaction capacity density and the discharge platform potential. Additionally, the conductivity of the polymer worsens and the electron transport rate slows down, which is not conducive to the expression of capacity at a high rate. However, by optimizing the polymerization position, the steric hindrance can be reduced to the greatest extent to improve the carbonyl activity, and the electronic conductivity can be improved by doping carbon materials with high conductivity.

### 3.4 Covalent Organic Frameworks (COFs)

Covalent organic skeleton polymer is a crystalline material with ordered porous structure formed by thermodynamically controlled reversible polymerization of light elements such as C/H/O/N/B. COFs have many properties, such as a high specific surface area, uniform pore size, high thermal stability, low density, and diverse structure.^161^, ^162^, ^163^ In ACCs, there is a highly crystalline COF structure in the highly reversible polymerization of imide and some amino groups.

In ACCs, the highly reversible reaction of imide with amino (amino quinones) has the most potential to form a stable COF structure. Among the molecules discussed at present, both PA‐10 and PI‐1‐21 have stable crystalline COF structures. Recently, Chen et al. reported a 2D microporous COF, poly(imide‐benzoquinone), formed via in situ polymerization on graphene (PI‐21) to function as a cathode material for LIBs. Such a structure favors charge transfer from graphene to PIBN and full access of both electrons and Li^+^ ions to the abundant redox‐active carbonyl groups. This enables large reversible specific capacities of 271.0 and 193.1 mAh g^−1^ at 0.1 and 10 C, respectively, and retention of more than 86 % after 300 cycles.^127^ The outstanding electrochemical properties of PI‐21 will lead to further studies on the design and general purpose of electrochemical COFs. However, at present, most of the molecules do not form the extended structure of COFs, and the COFs with high static specific surface areas can be synthesized by different thermodynamic controls.

## 4 Electrochemical Behavior of ACCs in Aqueous Solution

The dissolution of ACCs in liquid organic electrolytes, leading to low capacity and cycle life, is the largest obstacle. Improving the conductivity and binding in the active material, and the selection of a solid electrolyte and gel electrolyte, could alleviate the side effects. However, previous studies have shown that the ionic conductivity was reduced, and the processing conditions were cumbersome. To address the dissolution of ACCs, aqueous electrolytes have attracted research attention. The aqueous electrolytes were not self‐igniting, and the processing conditions were simple and cheap. More importantly, the ionic conductivity was two orders of magnitude higher than that of organic electrolyte, and a high power density could be easily achieved. Last but not least, in aqueous solution batteries, cationic aqueous solution batteries can be prepared and manufactured in a nondemanding atmosphere. Although aqueous solution batteries are still in their infancy, various cationic batteries in an aqueous electrolyte with nucleophilic addition reaction of the carbonyl are discussed, such as those of H^+^,^165^ NH_4_^+^,^65^ Li^+^,^166^, ^167^ K^+^,^168^, ^169^ Na^+^,^170^, ^171^, ^172^ Mg^2+^,^62^ Zn^2+^,^173^, ^174^, ^175^ and Ca^2+^.^63^ However, there are also many limitations of aqueous solution batteries that affect the electrochemical performance of ACCs. Moreover, since the aqueous solution batteries are still in the early stage of development, many types of cations have been studied, resulting in few research articles of single cation aqueous solution batteries. At present, the factors affecting ACCs in aqueous solution batteries are mainly the chemical stability and electrochemical stability caused by pH and solute concentration, which limits the application of ACCs as an electrode material.^176^ Therefore, using pH as a classification standard, the effects of ACC materials on batteries in different pH environments were investigated.

### 4.1 Acidic Aqueous Solution

The electrochemical system of ACCs in an acidic aqueous solution is unstable, and there are some side reactions of the active material, such as an irreversible chemical reaction that takes place between the electrolyte and the negative electrode. The first task was to determine whether hydrogen ions would participate in the nucleophilic addition reaction. The electrochemical mechanism of PA‐3 with hydrogen ion in an acidic solution was studied. The radius of hydrated hydrogen ions (100 ± 10 pm) is close to that of Na^+^ (102 pm).^165^ The shrinkage and expansion of the crystal structure of PA‐3 in acidic electrolyte during the charge–discharge process were characterized by in situ XRD, and it was found that the hydrated hydrogen ions could be embedded with the carbonyl group. PA‐3 exhibited a high reversible capacity of 68 mAh g^−1^ and a stable cyclability, with 85% capacity retention over 100 cycles at 1 A g^−1^. In acidic electrolytes, hydrogen ions would form a competitive relationship with metal cations, which would restrict the electrochemical performance of ACCs.

### 4.2 Alkaline Aqueous Solution

There were also side reactions between the active substances and the electrolyte in alkaline aqueous solutions. However, it is gratifying that quinone electrode materials could be used in alkaline electrolytes. Anders et al. compared the electrochemical performance of 2,6‐dihydroxyanthraquinone (DHAQ), oligomerized anthraquinones, oligo(vinylanthraquinone) (OVAQ), and oligo[benzene‐1,4‐dithiol‐alt‐(1,5‐dichloro‐anthraquinone)] (OBDTAQ).^177^ DHAQ could increase the potential of the whole cell for the donor hydroxyl group. The electron donating effect of the alkyl group was weaker than that of —S—; therefore, the application potential was in the order DHAQ, OBDTAQ, and OVAQ. Their *C*_initial_ values could reach the *C*_theoretical_. However, anthraquinone had high solubility, resulting in a rapid decay of the capacity of DHAQ, and potassium ions in the electrolyte solution entered the anode to balance the negative charge of the reduced anthraquinone, which caused volume expansion upon charging. The coupling of water molecules with potassium ions caused the expansion of the electrodes. As a result, electrical contact between the micro‐/nanoconductive graphite and the carbon black particles was lost, resulting in poor cycle performance of DHAQ and OVAQ (capacity after 100 cycles was only retained at 5–17%), whereas the OBDTAQ capacity was maintained at 45%. Improving the solubility of the quinone electrode materials and alleviating the volume change were the main ways to improve the electrochemical performance.^178^

### 4.3 Neutral Aqueous Solution

An aqueous solution with a pH of ≈7 is currently the most widely studied and promising for aqueous batteries. Compared with acidic/alkaline electrolytes, the side reaction of water in the neutral solution was in a relatively stable state (1.23 V), and the active material did not react with the electrolyte. However, low voltage in the neutral electrolyte is a major factor inhibiting the development of water batteries. In addition to improving the carbonyl activity by rationally designing the molecular structure, it was also possible to suppress the movement of free water using a high concentration of electrolyte and stabilizing the electrochemical window.^179^ In short, a neutral electrolyte relatively easily realized each cation compared with an acidic or alkaline aqueous solution.

PTCDA was used as a positive electrode material to investigate the electrochemical behavior of Mg^2+^ and Ca^2+^.^62^ During the discharge process, Mg^2+^ and oxygen anion combine to form an O—Mg bond. The larger Mg^2+^ embedded in PTCDA caused a shrinkage of the (011) plane of the crystal structure, and the expansion of the (021) plane revealed the reaction process. However, this resulted in a larger volume of Ca^2+^ and the collapse of the crystal form. The *C*_initial_ of PTCDA with Mg^2+^ was 125 mAh g^−1^ (1 C), and a good rate capability of 75 mAh g^−1^ (3.7 C) was maintained. The outstanding energy rate for the arrangement of Mg^2+^ and PTCDA indicated a “salmon” structure, which promoted the rapid transport of magnesium ions. When PTCDI was used as cathode material containing NH_4_^+^ in the electrolyte,^65^ the porous space structure of Ni‐APW provided a continuous ion channel for ammonium ions during the charge–discharge process. PTCDI exhibited a reversible capacity of 41 mAh g^−1^, an excellent cycle life over 1000 cycles with 67% capacity retention, and an average coulombic efficiency (CE) of ≈97.6%, with an outstanding rate performance (35 mAh g^−1^ at 10 C).

**Figure 11:**
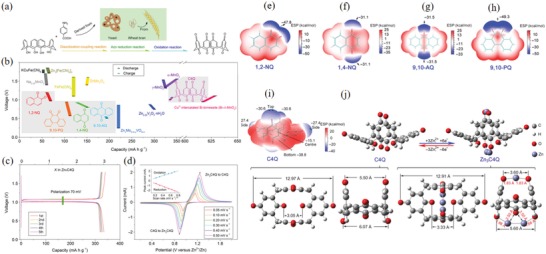
Quinone electrodes in aqueous ZBs. a) Schematic diagram for preparing C_4_Q. b) Discharge/charge voltages and capacities of selected quinone compounds (1,2‐NQ, 1,4‐NQ, 9,10‐PQ, 9,10‐AQ, and C_4_Q) in aqueous ZBs. The typically reported inorganic electrodes, including KCuFe(CN)_6_, Na_0.95_MnO_2_, Zn_3_[Fe(CN)_6_]_2_, FeFe(CN)_6_, ZnMn_2_O_4_, Zn*_x_*Mo_2.5+_ *_y_*VO_9+_ *_z_*, Zn_0.25_V_2_O_5_ · *n*H_2_O, g‐MnO_2_, a‐MnO_2_, and Cu^2+^‐intercalated Bi‐birnessite (Bi–d‐MnO_2_), are also listed for comparison. c) Galvanostatic discharge/charge curves of Zn–C_4_Q battery at 20 mA g^−1^. The upper *x*‐axis represents the uptake number of Zn ions. One Zn^2+^ with two‐electron transfers generates a specific capacity of 112 mAh g^−1^. d) Cyclic voltammetry (CV) curves of Zn–C_4_Q batteries at 0.05, 0.10, 0.20, 0.30, 0.40, and 0.50 mV s^−1^. The reduction and oxidation peaks are linked with arrows. The inset shows the corresponding linear fit of the peak current and the square root of the scan rate. Deducing the active sites and structure evolution of quinone electrodes. The ESP‐mapped molecular van der Waals surface of e) 1,2‐NQ, f) 1,4‐NQ, g) 9,10‐AQ, h) 9,10‐PQ, and i) C_4_Q. Surface local minima of ESP are represented as blue spheres, and the corresponding ESP values are marked out by numbers. j) Optimized configurations of C_4_Q before and after Zn ion uptake. Bottom: Corresponding configurations at different viewpoints. The distance between O–O and Zn–O has been labeled in angstroms. Reproduced with permission^173^ Copyright 2018, American Association for the Advancement of Science.

A series of small‐molecule quinones as electrode materials for ZIBs with aqueous solutions have been reported.^173^ The order of average *V*_platform_ from low to high was 9,10‐AQ, 9,10‐PQ, 1,4‐NQ, 1,2‐NQ, and C4Q. The *V*_platform_ values and capacities of the AQ series are compared in **Figure** **11**. C_4_AQ showed a pair of redox peaks with good reversibility and a good ion diffusion coefficient. The *C*_initial_ was 335 mAh g^−1^ (*C*_theoretical_ is 446 mAh g^−1^). The CEs of the first five cycles were ≈93%, and the potential difference of the charge–discharge platform was only 70 mV. Owing to the dissolution and ionization of the discharge product Zn*_x_*C_4_AQ, the dissolved C_4_AQ^2^ *^x^* ^−^ in the electrolyte would pass through the separator and produce a self‐discharge reaction, reducing the cycle life. A Nafion membrane, as a cation‐selective membrane, was used as an effective barrier, and the capacity at 1.5 C for 1000 cycles was retained at 87% of the *C*_initial_.

### 4.4 Coordination Theory in Aqueous Solution Batteries

Carbonyl compounds not only undergo nucleophilic addition reaction but also exhibit a coordination mechanism for cations with spacer orbitals. The cations act as central atoms that combine with oxygen in one or more ligands to form a coordination compound. The coordination reaction of several carbonyls in the aromatic carbonyl molecule with a cation can enhance the binding force and stabilize the molecular structure; the number of carbonyls is generally 2, 4, 5, or 6. This coordination mechanism can be used to explain the cycle stability of ACCs in polyvalent cationic batteries in aqueous solution. The coordination compounds formed by the coordination structure can be used to increase the storage charge space in organic batteries.^174^, ^175^, ^180^

**Figure 12:**
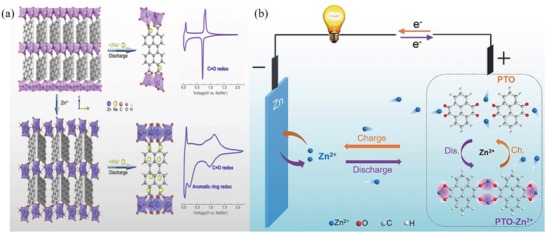
a) The behavior of Na^+^ storage in Zn‐PTCA. Reproduced with permission^180^ Copyright 2018, Elsevier. b) Illustration of the reversible reaction mechanism of the aqueous Zn‐PTO battery. Reproduced with permission^175^ Copyright 2018, Wiley‐VCH.

Zn^2+^ is a strong Lewis acid that can form stable coordination bonds with electron‐donating ligands. Furthermore, Zn^2+^ is redox inert, with an outer electronic orbital configuration of 3d^10^. The wavy layered 3D structure and the large interspace of zinc perylenetetracarboxylates (Zn‐PTCA) make it possible to form a fast diffusion channel for more insertion and extraction of Na^+^. Zn‐PTCA has a stretched space between adjacent perylene planes, which enables the activation of aromatic rings as sodium storage sites. Zn‐PTCDA can reversibly store 8 electrons (**Figure** **12**a).

In a ZIB with aqueous solution, the binding mechanism of pyrene‐4,5,9,10‐tetraone (PTO) and zinc ions is an ion coordination mechanism, and the stable coordination structure renders Zn‐PTO insoluble (Figure 12b). PTO shows a high specific capacity (336 mAh g^−1^) for Zn^2+^ storage with fast kinetics and high reversibility. PTO sustained a capacity retention of ≈70% and a delivered capacity of 145 mAh g^−1^ over 1000 cycles, along with a CE of ≈100%, expressing the durable cycle stability of the PTO cathode.

### 4.5 Conclusion

The decomposition potential of aqueous solution batteries limits the range of potential windows, while the unstable electrolyte environment can lead to unstable side reactions during cycling, such as water decomposition. Therefore, many ACCs are difficult to exert in aqueous solution batteries due to the potential window. It is apparent that the high solubility of carboxylates is not suitable for use in water batteries. Irreversible chemical reactions and inactivation reactions under strong acid and strong bases also hinder most ACCs. Therefore, the neutral environment is currently the most applicable aqueous solution electrolyte. In particular, imide and benzoquinone, which have been used in an organic electrolyte, also exhibit excellent electrochemical performance in an aqueous solution battery and are likely to be widely studied in various cationic aqueous solution batteries.

It is worth noting that the electrochemical reaction mechanisms of monovalent cations and polyvalent cations are different, indicating that the electrochemical reaction of active materials in aqueous solution batteries is not limited to nucleophilic addition reactions but can include the coordination theory of polyvalent cations. In short, the exploration of aqueous solution batteries is just beginning.

## 5 Summary: The Relationship between Structure and Performance

**Figure 13:**
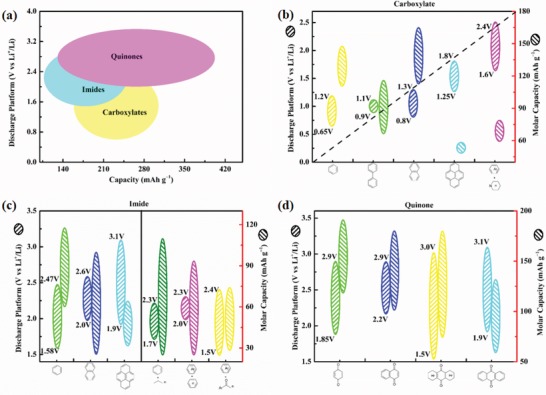
a) Main carbonyl compound platform potential and capacity range. b) Core structure and electrochemical properties of carboxylate. c) Imide core structure and electrochemical properties. d) Quinone core structure and electrochemical properties.

ACCs exhibit the advantages of adjustable structure and high capacity density, and they have great potential as battery materials for large‐scale energy storage. Rational molecule design, stable structures, multiple active sites, and multistep electrochemical reactions are key to improving the actual capacity density, *V*_platform_, cycle life, and rate performance. The mechanism of embedding/stripping carbonyl groups and cations has been analyzed. The essence of the electrochemical reaction of the carbonyl group is the nucleophilic addition reaction. The separation and binding of O–Li results in charge transfer. Therefore, the proper binding force and bond length of the Li—O bond keeps the binding and disengagement rate of Li and O relatively stable. To maintain good reversibility of ACCs when charging and discharging, the exchange current density, *R*_ct_, and other electrochemical parameters have been optimized. The relationship between the molecular structure and the electrochemical properties is summarized in **Figure** **13**. It has been shown that the molecular design and structure diversification directly determine the electrochemical performance. The activation energies for nucleophilic addition reactions of ACCs, from low to high, were in the order of quinone, imide, and carboxylate. Carboxylates exhibit inherent solubility inertia, and their core structure has a large influence on the *V*_platform_ and capacity density. It was shown that a larger π structure leads to a higher *V*_platform_ and a lower capacity density. Heterocyclic carboxylates exhibit a higher discharge plateau voltage and better cyclic life (Figure 13b). The impact of π structure in imides and quinones increases the capacity density. It is clear that there is a contradiction between the stability of the molecular structure and the increase in capacity density due to equilibrium of the π structure (Figure 13c,d). The additional π structure with an active center stabilizes the molecular structure and alleviates the loss in capacity. The inherent insulation of ACCs can improve the electronic conductivity and the ion diffusion rate through the 3D molecular structure.

This comprehensive analysis of the research progress and reaction mechanisms of ACCs in organic/water batteries leads to the following conclusions. 1) The nucleophilic addition mechanism and ion coordination theory will become important theories when investigating the reaction mechanisms between carbonyl compounds and cations. 2) ACC materials in organic LIBs, the molecular structure with the most future potential, is similar to TP‐6/PI‐4/AQ‐20/PQ‐15, with more highly active carbonyl groups and stable molecular structures, while simultaneously maintaining good electrochemical stability. 3) Combined with the polarity of carboxylic acid substituents, carboxylate can be a good negative electrode material. The combination of imide and quinones to form COFs can not only provide stable chemical structure and sufficient reaction sites, it can also contribute to the site of lithium storage. This is a new type of ACC electrode material and has great potential. 4) The development of aqueous solution batteries is far less perfect and comprehensive than the development of organic phases, but it is foreseeable that in the future of aqueous solution batteries, quinone and imide will be widely used in various cationic aqueous solution batteries. Among these, the electrochemical performance and the ionic radius of the cation has a strong relationship. 5) Regarding the industrial application of ACCs, there are two urgent problems to be solved. First, finding simple, efficient, and cheap methods to obtain active substances from natural sources and biomass and the design of economical and mature industrial processes are key for commercialization. Second, it is necessary to improve the capacity density, coulomb efficiency, and activity utilization ratio of the high‐load electrodes. 6) ACCs are used as active substances instead of inorganic materials for battery energy storage. This is in line with the concept of sustainable development and they can be widely used in portable electronic devices, storage systems, and transportation. Further promotion of commercialization is important for solving the current energy crisis and addressing environmental pollution.

The reversible dissociation and recombination in the nucleophilic addition reaction of the carbonyl group is due to the oxidation–reduction mechanism. Structural molecules similar to carbonyl groups, such as N=N, N=O, P=O, and S=O, are also electrochemically active, which is worth exploring in the future. Although ACCs, as electrode active materials, have been extensively studied, their industrial production has not yet been achieved, and solubility problems may the leading factor in their future research. In this paper, the relationship between the molecular structure and electrochemical properties of ACCs in organic and aqueous electrolytes was summarized. Through the modification of molecular structure and atomic size to improve the inherent solubility and conductivity of ACCs, the most suitable solutions for different types of ACCs were proposed. These rules can guide the design of corresponding carbonyl compounds. It is expected that in the near future, after solving the challenge of high load and low loss of ACCs and finding a simple and efficient industrial route for the large‐scale synthesis of ACCs, the application of ACCs in organic and aqueous solutions will achieve large‐scale production and gradually replace inorganic electrodes to become the next generation of major energy storage materials. In the future, they may become widely used in electric energy vehicles, portable electronic devices, and in other green energy applications.

## Conflict of Interest

The authors declare no conflict of interest.
